# Identifying the role of NUDCD1 in human tumors from clinical and molecular mechanisms: a study based on comprehensive bioinformatics and experimental validation

**DOI:** 10.18632/aging.204813

**Published:** 2023-06-19

**Authors:** Bin Han, Jinsong He, Qing Chen, Min Yuan, Xi Zeng, Yuanting Li, Yan Zeng, Meibo He, Dan Feng, Daiyuan Ma

**Affiliations:** 1 GCP Center/Institute of Drug Clinical Trials, Affiliated Hospital of North Sichuan Medical College, Nanchong, China; 2 Department of Pharmacy, Affiliated Hospital of North Sichuan Medical College, Nanchong, China; 3 Institute of Pharmacy, North Sichuan Medical College, Nanchong, China; 4 Department of Gastroenterology, Affiliated Hospital of North Sichuan Medical College, Nanchong, China; 5 Department of Oncology, Affiliated Hospital of North Sichuan Medical College, Nanchong, China

**Keywords:** NUDCD1, pan-cancer, prognosis, immune, mechanisms

## Abstract

NUDCD1 (NudC domain-containing 1) is abnormally activated in multiple tumors and has been identified as a cancer antigen. But there is still no pan-cancer analysis available for NUDCD1 in human cancers. The role of NUDCD1 across multiple tumors was explored using data from the public databases including HPA, TCGA, GEO, GTEx, TIMER2, TISIDB, UALCAN, GEPIA2, cBioPortal, GSCA and so on. Molecular experiments (e.g., quantitative real-time PCR, immunohistochemistry and western blot) were conducted to validate the expression and biological function of NUDCD1 in STAD. Results showed that NUDCD1 was highly expressed in most tumors and its levels were associated with the prognosis. Multiple genetic and epigenetic features of NUDCD1 exist in different cancers. NUDCD1 was associated with expression levels of recognized immune checkpoints (anti-CTLA-4) and immune infiltrates (e.g., CD4+ and CD8+ T cells) in some cancers. Moreover, NUDCD1 correlated with the CTRP and GDSC drug sensitivity and acted as a link between chemicals and cancers. Importantly, NUDCD1-related genes were enriched in several tumors (e.g., COAD, STAD and ESCA) and affected apoptosis, cell cycle and DNA damage cancer-related pathways. Furthermore, expression, mutation and copy number variations for the gene sets were also associated with prognosis. At last, the overexpression and contribution of NUDCD1 in STAD were experimentally validated *in vitro* and *in vivo*. NUDCD1 was involved in diverse biological processes and it influenced the occurrence and development of cancers. This first pan-cancer analysis for NUDCD1 provides a comprehensive understanding about its roles across various cancer types, especially in STAD.

## INTRODUCTION

Tumorigenesis and the biological mechanisms underlying tumor initiation, progression and metastases are complex and incompletely understood [1–4]. Public platforms that disseminate cancer genetic information such The Cancer Genome Atlas (TCGA) program [5], Gene Expression Omnibus (GEO) database [6] and the Encyclopedia of DNA Elements (ENCODE) project [7] provide functional genomic data for the numerous types of human tumors. A systematic pan-cancer analysis of the data in these repositories can assist to link genes and clinical prognosis and thus facilitate the identification of molecular mechanisms of cancer progression [8, 9].

NUDCD1 (NudC domain-containing 1 also known as CML66 or OVA66) is a 66 kDa protein abnormally activated in multiple tumors and has been identified as a cancer antigen [10]. The gene encoding NUDCD1 possesses several isoforms derived from 12 alternatively-spliced exons and is overexpressed in numerous human tumor tissues [11]. NUDCD1 functions are involved in diverse biological processes and especially the epithelial-mesenchymal transition (EMT) [12] and it can also trigger multiple tumors signaling pathways to induce a complex and integrated phenotype that affects cell proliferation, migration, invasion and apoptosis [13]. NUDCD1 has been functionally linked to tumorigenesis in pancreatic, lung, colorectal, ovarian and cervical cancer [12, 14–16].

The functions of NUDCD1 in these cancers have been demonstrated via cell and animal-based evidence but a clinical data-based pan-cancer analysis has not been conducted. In the current study, for the first time, we used the data from public platforms to conduct a pan-cancer analysis of NUDCD1 to understand the role of NUDCD1 in carcinogenesis. This pan-cancer analysis utilized multiple components including expression patterns, prognosis and survival, DNA methylation, genetic and epigenetic features, immune system and infiltration, drug sensitivity as well as enrichment analysis. Importantly, we verified the role of NUDCD1 in STAD using clinical samples and gastric cell lines.

## RESULTS

### Expression patterns of NUDCD1 in normal tissues and cells

**Figure 1:**
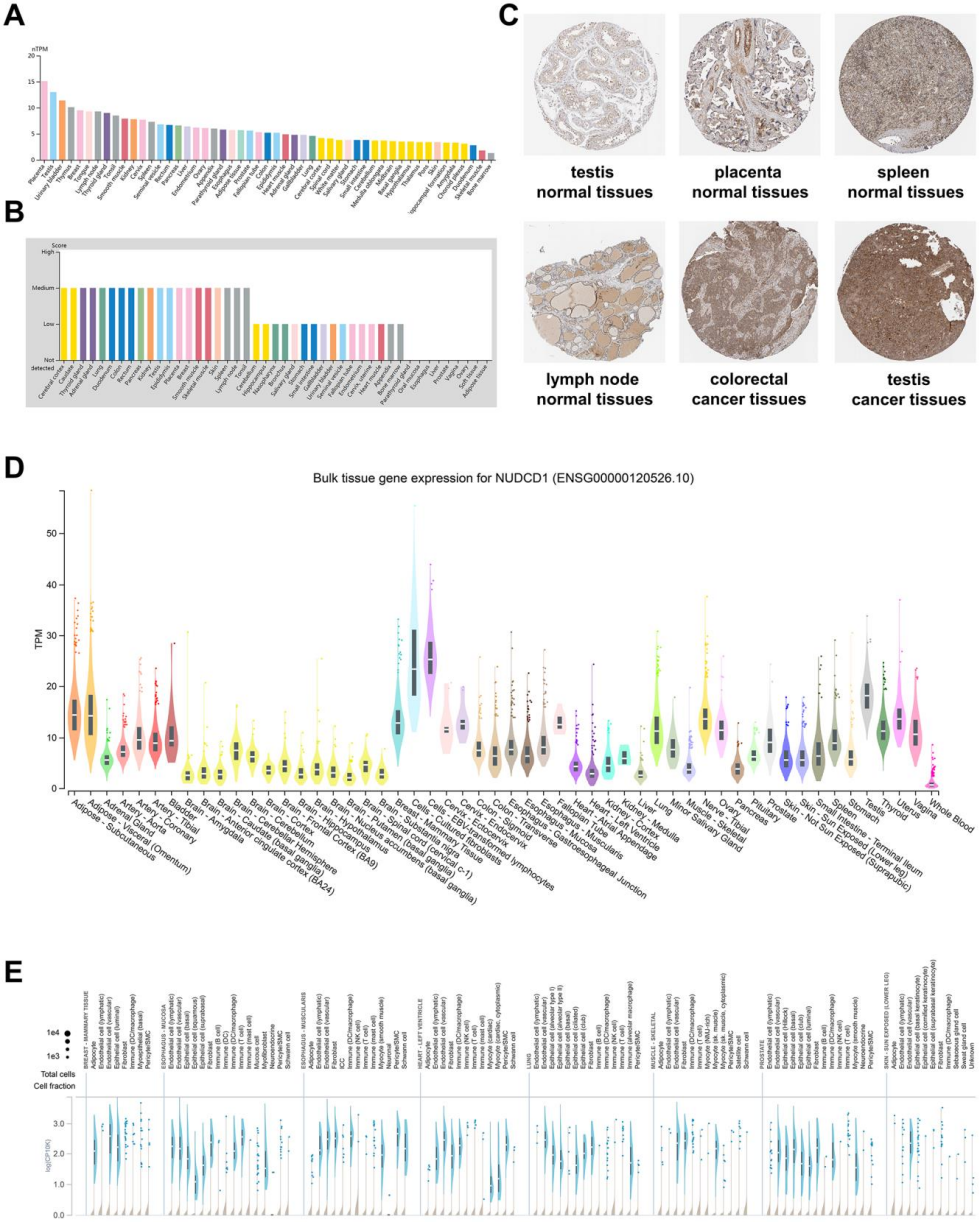
**NUDCD1 expression profiles in human normal and cancerous tissues.** (**A**) NUDCD1 expression levels in human tissues based on Internally generated Human Protein Atlas (HPA) RNA-seq data. (**B**) Protein expression levels of NUDCD1 in human tissues. Protein expression data is shown for each of the 44 tissues from the HPA database. (**C**) IHC images of NUDCD1 in normal testis tissues, normal placenta tissues, normal spleen tissues, normal lymph node tissues, colorectal cancer tissues, and testis cancer tissues from HPA. (**D**) Bulk tissue gene expression and (**E**) single tissue expression for NUDCD1 based on the GTEx database.

To detect the mRNA and protein expression profiles of NUDCD1 in human normal and cancer tissues, the NUDCD1 expression data for normal tissues were evaluated using the HPA database. An overview of NUDCD1 mRNA and protein expression data indicated that NUDCD1 distribution possessed only a low tissue specificity (Supplementary Figure 1A). NUDCD1 exhibited high mRNA expression levels in placenta, testis and urinary bladder (Figure 1A). The NUDCD1 mRNA expression in GTEx (Genotype-tissue expression), FANTOM5 (Function annotation of the mammalian genome 5) and consensus dataset (dataset created by combining the HPA and GTEx) was shown in Supplementary Figure 1B–1D. NUDCD1 protein expression was widely expressed with medium-low levels in most normal tissues (Figure 1B) and was primarily located in the nucleoplasm and cytosol (Figure 1C). Moreover, NUDCD1 was expressed in several cancers including testicular and colorectal cancer (Figure 1C). The bulk tissue gene expression from GTEx analysis revealed that NUDCD1 was highly expressed in cultured fibroblasts and EBV-transformed lymphocytes but expressed at only low levels in whole blood (Figure 1D). Single cell snRNA-seq single tissue expression for NUDCD1 was shown in Figure 1E. NUDCD1 possessed 4 isoforms (ENST00000521439.1, ENST00000427660.6, ENST00000519607.5 and ENST00000239690.8) differentially expressed in tissues (Supplementary Figure 1E) and the junctions and exon expression levels were defined for these isoforms (Supplementary Figure 2A, 2B). NUDCD1 immune cell expression indicated a low cell type-specificity (Supplementary Figure 2C–2E) and NUDCD1 expression was elevated in extracellular vesicles found in urine and lowest in the glomerular basement membrane (Supplementary Figure 2F).

### Expression patterns of NUDCD1 in cancer tissues and cells

**Figure 2:**
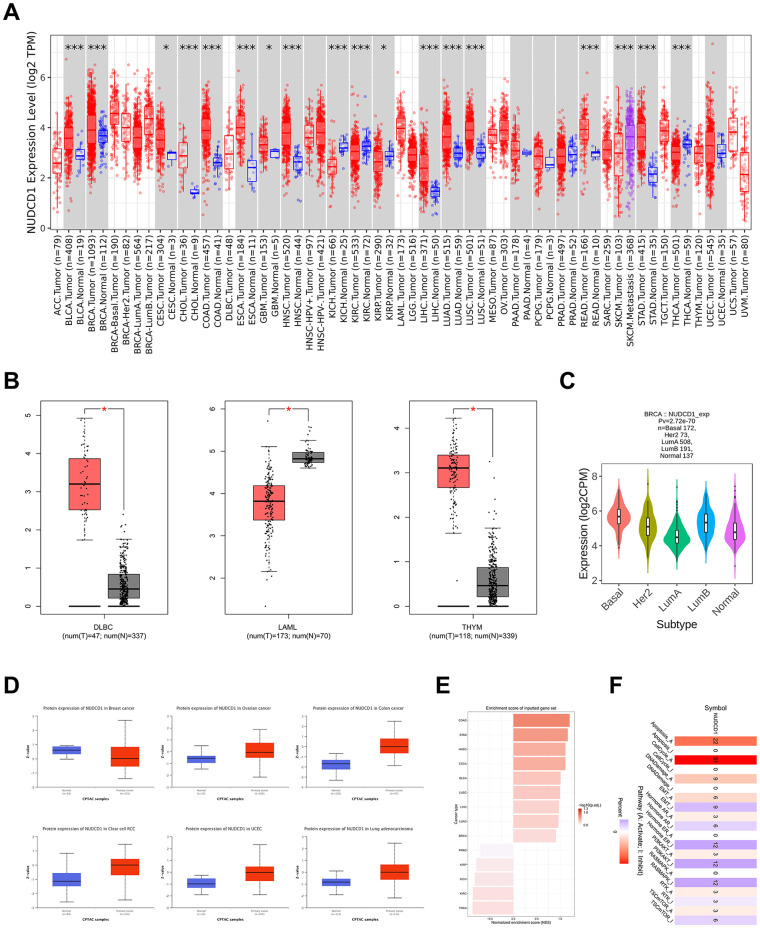
**NUDCD1 expression levels in different types of human cancers.** (**A**) NUDCD1 expression levels in different cancers and specific cancer subtypes from TIMER2. (**B**) NUDCD1 expression levels in DLBC, LAML and THYM (data from GEPIA2). (**C**) Expression status of the NUDCD1 gene in BRCA subtypes (data from TISIDB). (**D**) Expression levels of NUDCD1 total protein in breast cancer, ovarian cancer, colon cancer, clear cell RCC, UCEC and lung adenocarcinoma (data from UALCAN). (**E**) Enrichment scores for NUDCD1 in different cancers (data from GSCA). (**F**) Pathways affected by NUDCD1 mRNA expression (data from GSCA).

NUDCD1 has been previously defined as a cancer antigen and is abnormally upregulated in multiple tumors and acts as an oncogene [10]. NUDCD1 expression level was elevated in tumor tissues compared with the corresponding normal counterparts for BLCA (Bladder urothelial carcinoma), BRCA (Breast invasive carcinoma), CESC (Cervical squamous cell carcinoma and endocervical adenocarcinoma), CHOL (Cholangio carcinoma), COAD (Colon adenocarcinoma), ESCA (Esophageal carcinoma), GBM (Glioblastoma multiforme), HNSC (Head and Neck squamous cell carcinoma), LIHC (Liver hepatocellular carcinoma), LUAD (Lung adenocarcinoma), LUSC (Lung squamous cell carcinoma), READ (Rectum adenocarcinoma) and STAD (Stomach adenocarcinoma) (Figure 2A). NUDCD1 expression was abnormally expressed in DLBC (Lymphoid neoplasm diffuse large B-cell lymphoma), LAML (Acute myeloid leukemia), THYM (Thymoma) compared with normal tissue (Figure 2B) and was also present in BRCA subtypes (Figure 2C). NUDCD1 total protein was elevated in ovarian, colon, clear cell RCC (Renal cell carcinoma), UCEC (Uterine corpus endometrial carcinoma) and LUAD compared with normal tissues (Figure 2D). The enrichment score rankings (Figure 2E) were then associated with pathways effected by NUDCD1 (Figure 2F).

Tissue-based RNA expression can be affected by tumor heterogeneity or individual patient differences so we further analyzed NUDCD1 RNA expression in different cell lines. NUDCD1 was highly expressed in the cells from meningioma and CML (Chronic myelogenous leukemia), and low in the cells from chondrosarcomas and giant cell tumors (Supplementary Figure 3A). Immunofluorescence microscopy confirmed that the NUDCD1 protein was primarily localized to the nucleoplasm (Supplementary Figure 3B). NUDCD1 mRNA expression and copy numbers were linearly correlated (Spearman 0.48, p<0.01, Supplementary Figure 3C) while there was no statistically significant correlation between NUDCD1 mRNA and DNA methylation (RRBS) (p = 0.847, Supplementary Figure 3D). The overall expression of NUDCD1 in cell lines indicated that mesenchymal cells expressed the highest levels of NUDCD1 mRNA (Supplementary Figure 3E). We examined the effect of NUDCD1 expression on the pathological stages of cancers from the “Pathological Stage Plot” module of GEPIA2, TISIDB (web portal for tumor and immune system interaction), and GSCA (Supplementary Figure 4A–4C), that could also correlate NUDCD1 expression to tumor pathologic stage for KICH (Kidney chromophobe), KIRP (Kidney renal papillary cell carcinoma) and LUAD (Supplementary Figure 4D). NUDCD1 mRNA expression in tumor tissues was significantly higher than that in normal tissues for in BRCA, BLCA, HNSC, LUSC, ESCA, LUAD, LIHC (Liver hepatocellular carcinoma), STAD and COAD but not THCA (Thyroid carcinoma) (Supplementary Figure 5A). Subtype analysis indicated associations between NUDCD1 expression and immune or molecular subtypes for BRCA, LUAD and UCEC (Supplementary Figure 5B, 5C) and subtypes could be grouped between high and low NUDCD1 expression (Supplementary Figure 5D). Moreover, NUDCD1 expression was also positively correlated to tumor grades in CESC, LGG (Brain lower grade glioma), LIHC and UCEC and negatively correlated to STAD tumor grade (Supplementary Figure 5E).

### Prognostic significances of NUDCD1 in different cancers

**Figure 3:**
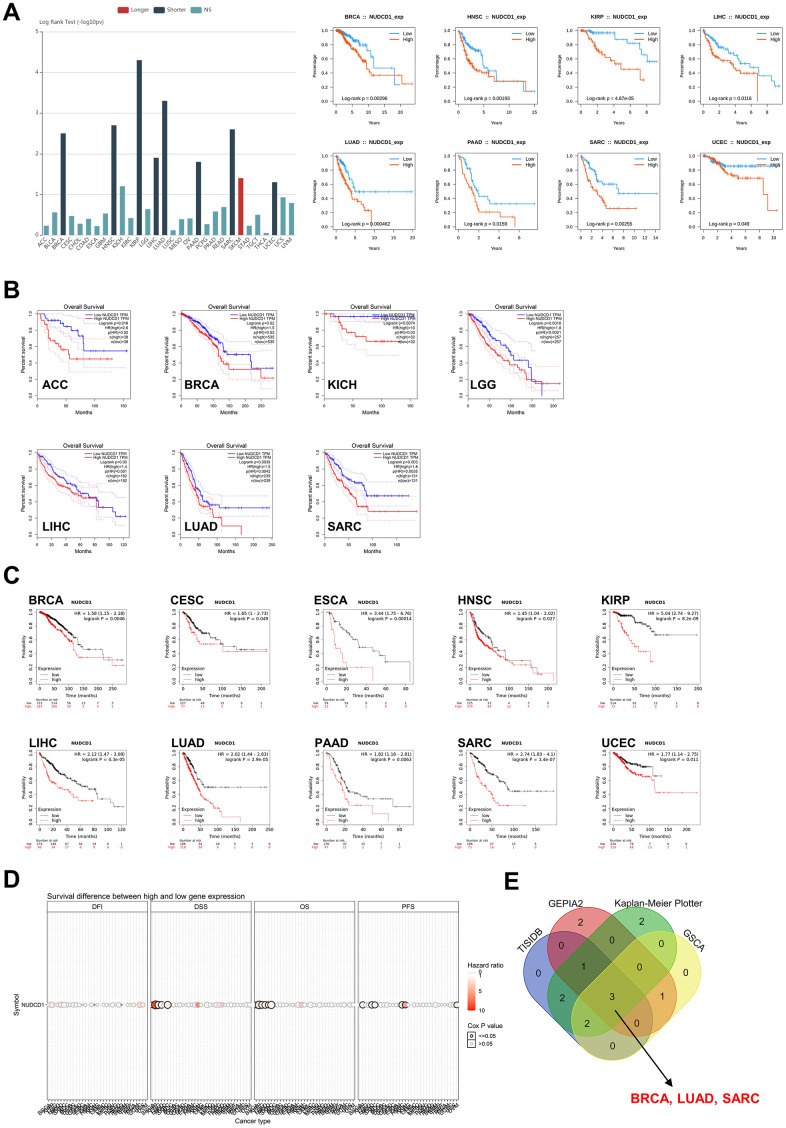
**Correlation between NUDCD1 expression and overall survival prognosis of cancers.** (**A**) Associations between NUDCD1 expression and overall survival across human cancers from TISIDB. (**B**) Associations between NUDCD1 expression and overall survival across specific human cancers from GEPIA2. (**C**) Effects of NUDCD1 expression on overall survival in multiple cancer types (data from Kaplan-Meier Plotter). (**D**) Survival differences between high and low NUDCD1 expression groups in multiple cancers (data from GSCA). (**E**) Wayne diagram summarizing the overall survival data from TISIDB, GEPIA2, Kaplan-Meier Plotter and GSCA.

NUDCD1 is aberrantly expressed in numerous tumor tissues and cells so we also explored the prognostic relevance of NUDCD1 in cancers. First, analyses based on TISIDB revealed that high expression of NUDCD1 indicated a shorter OS (overall survival) in 8 types of cancers (Figure 3A). Data from GEPIA2 suggested that NUDCD1 expression was correlated with OS in 7 types of cancers (Figure 3B). Moreover, Kaplan-Meier Plotter (KM Plotter) was used to draw overall survival curves of cancer patients (Figure 3C) and the OS from GSCA indicated distinct survival differences between high and low NUDCD1 expression groups in multiple cancers (Figure 3D). The combined results of above 4 databases indicated that in BRCA, LUAD and SARC (Sarcoma), increased expression of NUDCD1 predicted a poor overall survival (Figure 3E).

Immunohistochemistry data from HPA indicated that the NUDCD1 protein was more highly expressed in tumor samples compared to normal tissues in BRCA, LUAD and SARC (Supplementary Figure 6A). Regrettably, prognostic analysis of the NUDCD1 signatures in these 3 cancers revealed that its expression had weak to moderate implications for 1, 3, 5-year survival (Supplementary Figure 6B–6D). We also calculated disease-free survival (DFS) (Supplementary Figure 7A) and relapse-free survival (RFS) (Supplementary Figure 7B). We found a significant effect of NUDCD1 expression on DSS (disease-specific survival), DFI (disease-free interval) and PFS (progression-free survival) (Supplementary Figure 7C). Increased expression of NUDCD1 was a predictor of a poor DFS especially in SARC.

### The genetic and epigenetic features of NUDCD1 in cancers

**Figure 4:**
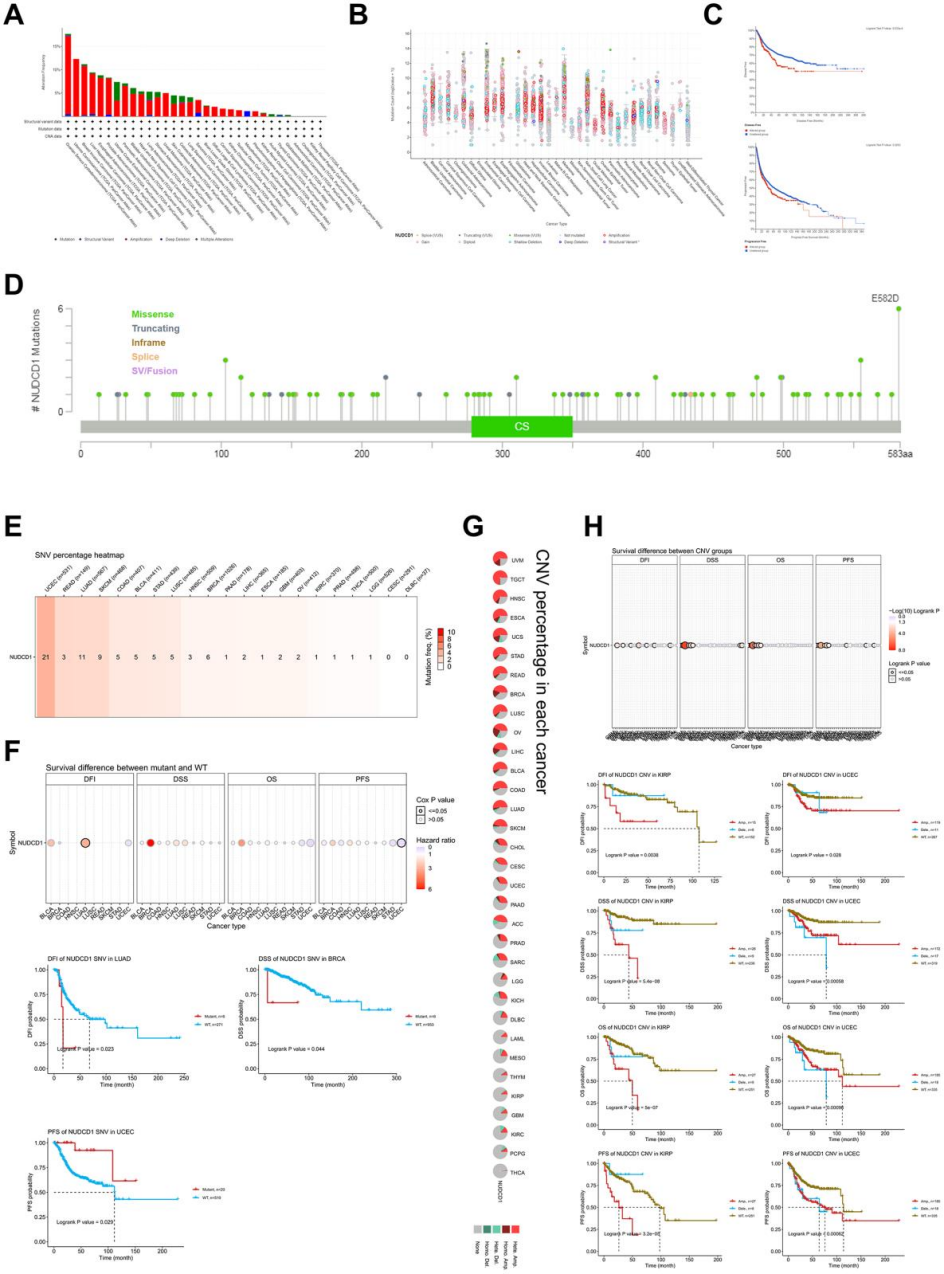
**Genetic and epigenetic features of NUDCD1 in different tumors.** (**A**) Alteration frequencies of NUDCD1 across different tumors from cBioPortal. (**B**) General mutation counts of NUDCD1 in various TCGA cancer types from cBioPortal. (**C**) Disease free survival (DFS) and progression free survival (PFS) between mutant and WT NUDCD1 in human cancers. (**D**) Mutation types and sites of NUDCD1 from cBioPortal. (**E**) SNV (Single Nucleotide Variation) of NUDCD1 in human cancers. The heatmap summarizes the frequency of deleterious mutations (data from GSCA). (**F**) Survival differences between mutant and WT NUDCD1 in human cancers (data from GSCA). (**G**) CNV (Copy Number Variation) of NUDCD1 in each cancer type. A global profile for heterozygous/homozygous CNV of NUDCD1 in each cancer (data from GSCA). (**H**) Survival differences between CNV and WT NUDCD1 in each cancer type (data from GSCA).

Genetic alterations of NUDCD1 in different tumor samples indicated that the highest alteration frequency (>15%) appeared for patients with ovarian serous cystadenocarcinoma. In contrast, low NUDCD1 alteration frequencies were detected with adrenocortical cancer, cholangiocarcinoma, kidney renal papillary cell carcinoma and thymoma. The primary type for these mutations was gene amplification (Figure 4A) and the general mutation type, structural variants and copy numbers in TCGA cancer types also varied (Figure 4B). Survival analysis revealed that cancer patients with a mutation in NUDCD1 had a significantly worse DFS and PFS than the patients without NUDCD1 mutations (Figure 4C) and there was no significant difference between DSS and OS (Supplementary Figure 8A).

A total of 103 mutation sites for the gene were identified, including 80 missense, 16 truncations, 2 splice and 5 fusion mutations between codons 1 and 583 (Figure 4D). In addition, “Single Nucleotide Variation” (SNV) (Supplementary Figure 8B) and “Copy Number Variation” (CNV) (Supplementary Figure 8C, 8D) were found that included 32 cancer types. There were also significant correlations of NUDCD1 CNV with its mRNA expression in most types of cancers with the exception of THCA, LAML and DLBC (Supplementary Figure 8E). The SNV percentage heatmap and CNV percentage in each cancer was displayed in Figure 4E, 4G, respectively. The highest SNV mutation frequency of NUDCD1 (21%) appeared in patients with UCEC while there were none found for patients with CESC and DLBC (Figure 4E). Survival differences between mutant and WT for SNV indicated a significantly worse DFI profile for LUAD, DSS in BRCA and PFS in UCEC (Figure 4F). Moreover, in KIRP and UCEC, NUDCD1 CNV was significantly worse for DFI, DSS, OS and PFS (Figure 4H).

### Methylation of NUDCD1 in cancers

**Figure 5:**
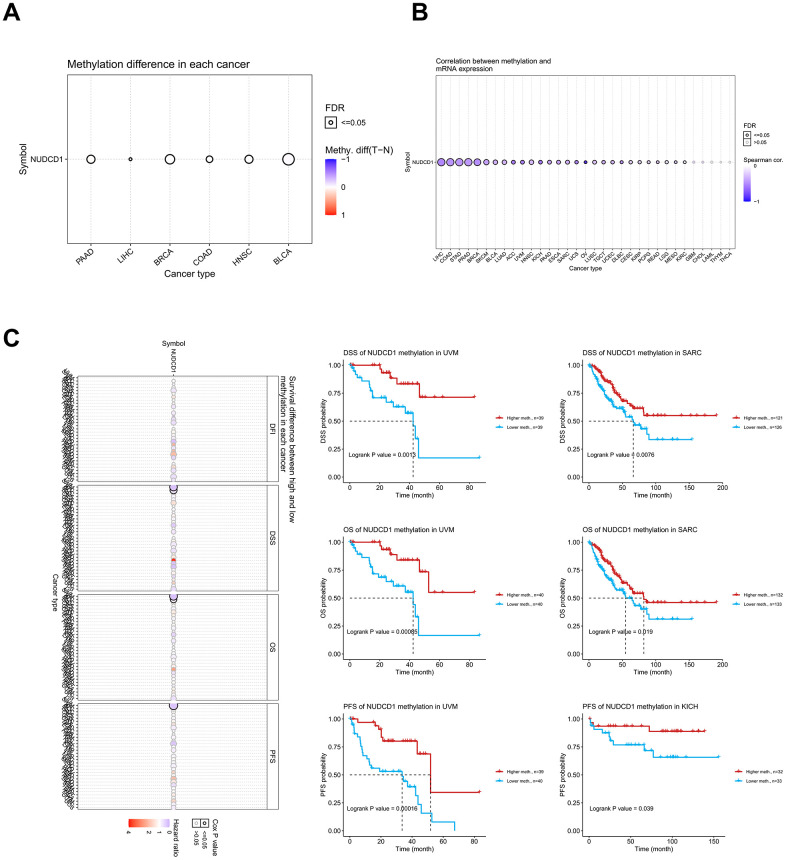
**Methylation analysis of NUDCD1 in different cancer types.** (**A**) Methylation differences between tumor and normal samples of NUDCD1 (data from GSCA). (**B**) Correlations between methylation and mRNA expression of NUDCD1 in the specific cancers (data from GSCA). (**C**) Survival differences between high and low methylation of NUDCD1 in specific cancers (data from GSCA).

DNA methylation of genes also plays a key role in the regulation of cancer progress and we used the GSCA database to explore the DNA methylation of NUDCD1 in pan-cancers. Methylation differences between tumor and normal samples occurred for BLCA, BRCA, COAD, HNSC, LIHC and PAAD (Pancreatic adenocarcinoma) (Figure 5A). DNA methylation of NUDCD1 was significantly and negatively correlated with its mRNA expression in most cancers with the exception of GBM, CHOL, LAML, THYM and THCA (Figure 5B). UVM (Uveal melanoma) patients with NUDCD1 methylation had better DSS, OS and PFS than the patients without methylation. Similarly, SARC patients with NUDCD1 methylation had a better prognosis for DSS and OS while KICH patients with NUDCD1 methylation possessed a greater PFS (Figure 5C). Clinical data and expression, copy number and DNA methylation data for UVM, SARC and KICH are depicted in Supplementary Figure 9A–9C.

### Interactions between tumor-immune system and NUDCD1

**Figure 6:**
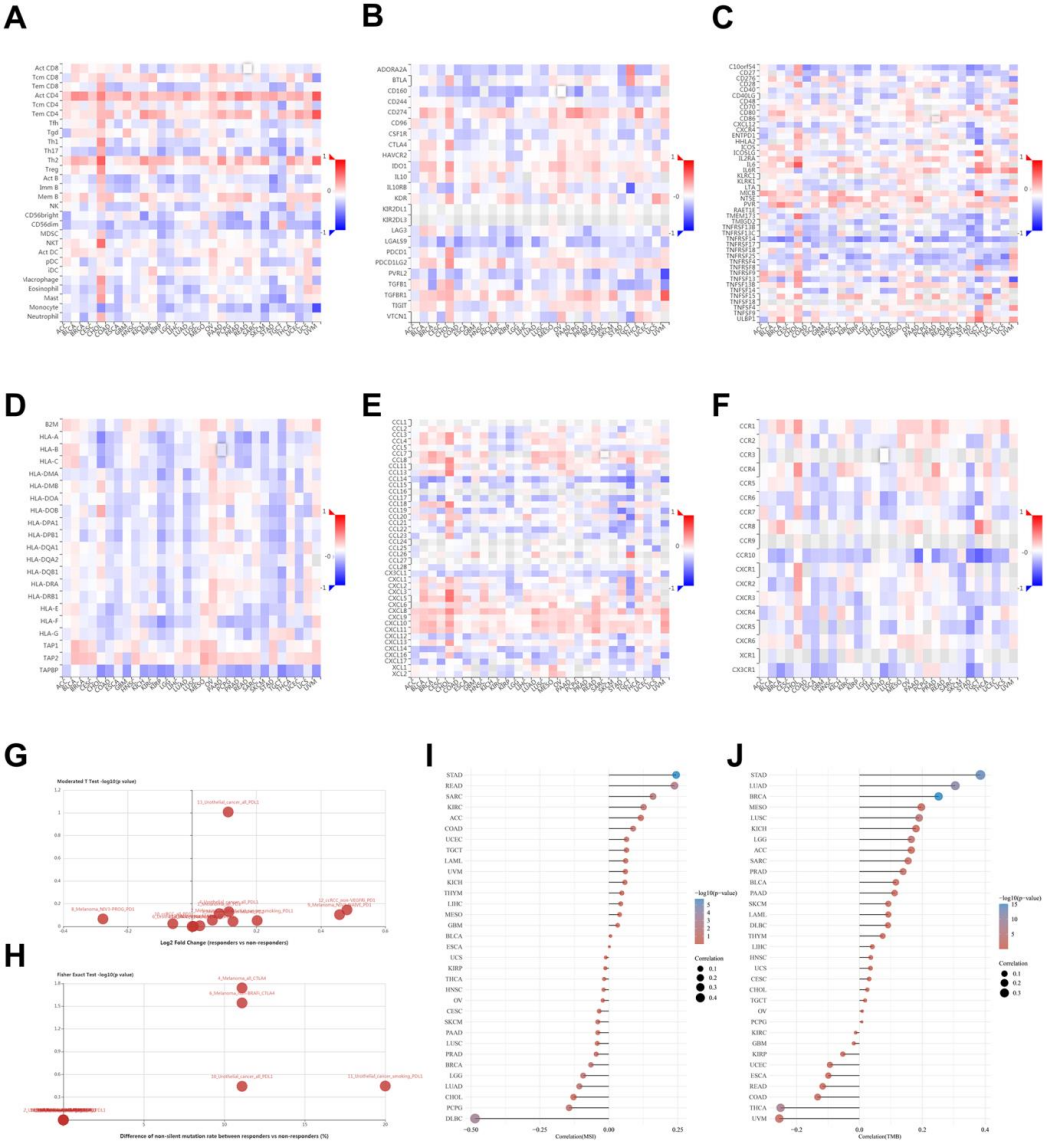
**Relationships between tumor - immune system and NUDCD1 expression.** Correlations between expression of NUDCD1 and (**A**) TILs (**B**) immunoinhibitors (**C**) immunostimulators (**D**) MHC (**E**) chemokines and (**F**) receptors across human cancers (data from TISIDB). (**G**) Expression and (**H**) mutation differences for NUDCD1 between responders and non-responders (data from TISIDB). Correlations between (**I**) MSI and (**J**) TMB and NUDCD1 mRNA expression levels in various cancers in the TCGA.

The interaction between tumors and the immune system plays a crucial role in cancer initiation, progression, and treatment. Therefore, elucidation of tumor and immune cell interplay would assist both the prediction of immunotherapy responses and the development of novel immunotherapy targets. TISIDB is a web portal for tumor and immune system interaction and integrates multiple heterogeneous data types. This portal was used to identify potential correlations between NUDCD1 expression and TILs, immunoinhibitors, immunostimulators, MHC, chemokines and receptors (Figure 6A–6F). It was found that NUDCD1 expression was not significantly different between responders and non-responders (Figure 6G). However, NUDCD1 did possess significant mutation differences between responders and non-responders for anti-CTLA-4 (ipilimumab) therapy for melanoma (Figure 6H).

The mismatch repair (MMR) pathway plays a critical role in identifying and repairing mismatched bases during DNA replication and genetic recombination [17]. It was found that NUDCD1 expression was positively correlated with MSI (microsatellite instability) in STAD while negatively correlated with MSI in DLBC (Figure 6I). NUDCD1 expression was positively correlated with TMB (tumor mutation burden) in STAD, LUAD and BRCA while negatively correlated with TMB in UVM and THCA (Figure 6J).

### Interactions between NUDCD1 and immune infiltration in cancer

**Figure 7:**
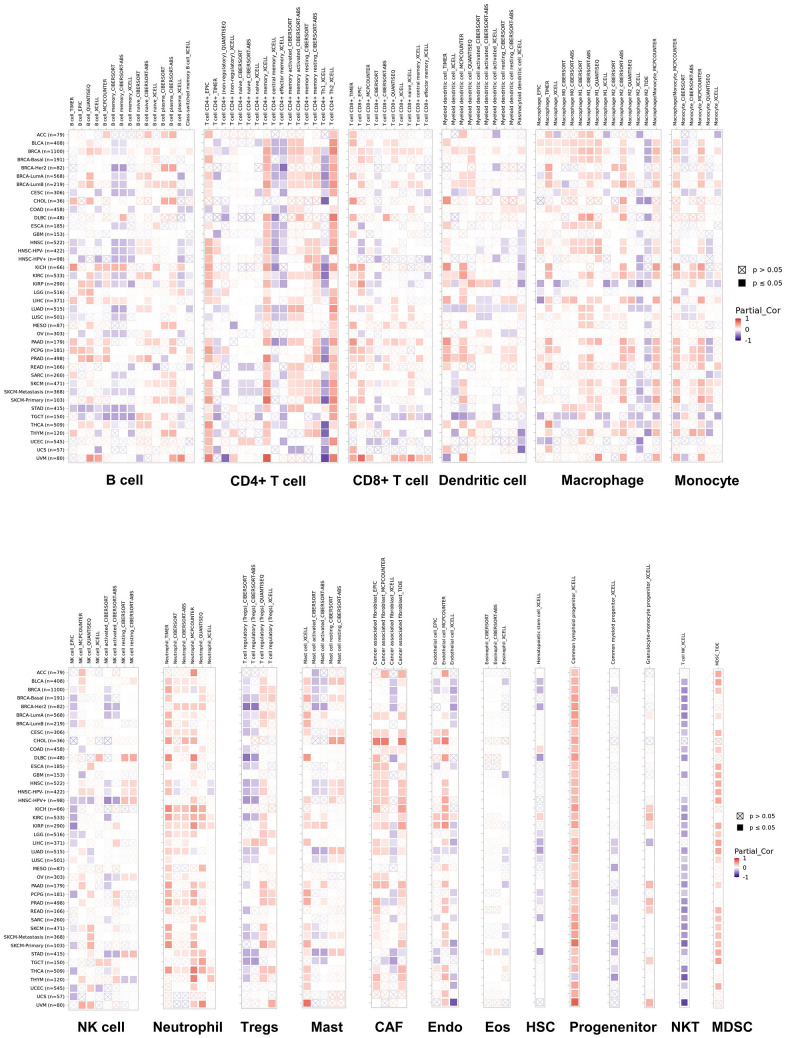
Correlations of NUDCD1 expression and immune infiltration in cancers (data from TIMER2).

To explore whether NUDCD1 relates to the process of immune infiltration in cancers, TIMER2 was employed to examine NUDCD1 expression in tumor-immune infiltrates. Overall, its gene expression was positively correlated with immune infiltrating levels of CD4+ and CD8+ T cells, macrophages, neutrophils, CAP, common myeloid progenitor X cells and MDSC (Figure 7). The negative and positive Spearman correlations between NUDCD1 expression and immune infiltrates are presented respectively in Supplementary Figure 10A, 10B. We also explored relationships between NUDCD1 methylation and immune infiltrates (Supplementary Figure 10C, 10D) and identified differences of immune infiltration between mutant and WT NUDCD1 in specific cancers (Supplementary Figure 10E) and correlations between NUDCD1 CNV and immune infiltrates (Supplementary Figure 10F, 10G).

### Correlations between NUDCD1 expression and drug sensitivity in cancers

**Figure 8:**
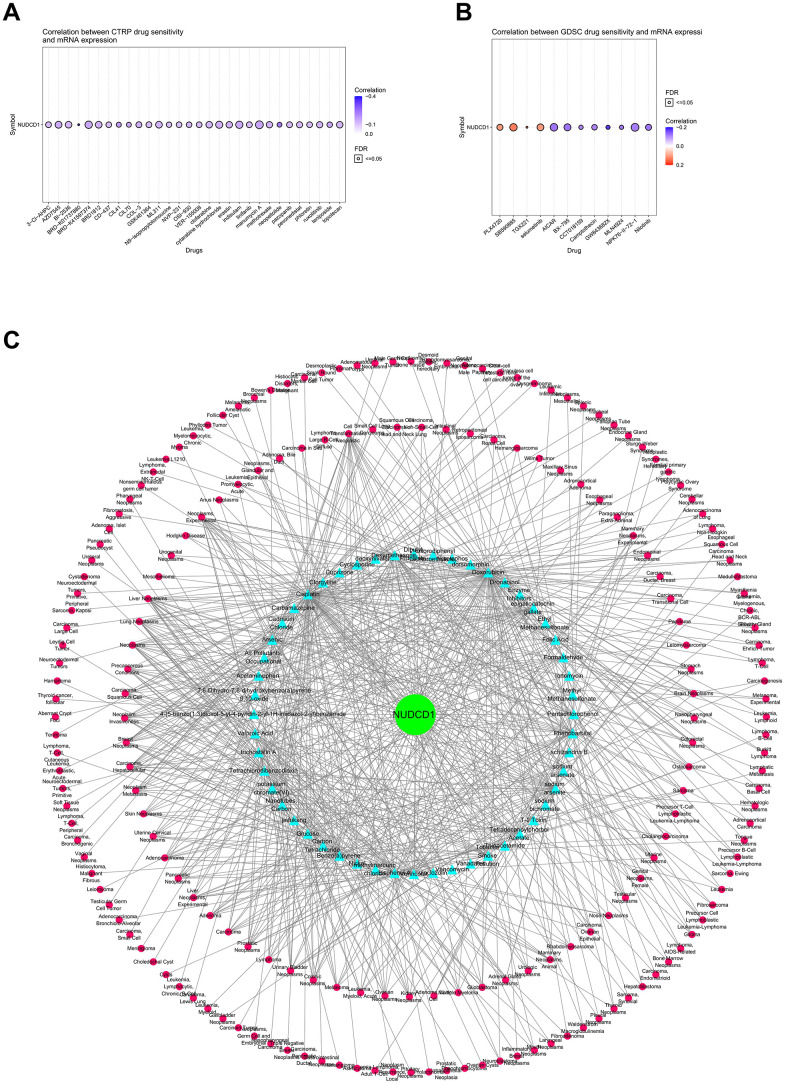
**Correlations between NUDCD1 expression and drug sensitivity in cancers.** (**A**) Correlations between NUDCD1 expression and the sensitivity of CTRP drugs (top 30) in pan-cancer (data from GSCA). (**B**) Correlations between NUDCD1 expression and the sensitivity of GDSC drugs (top 30) in pan-cancer (data from GSCA). (**C**) Associations between NUDCD1, chemicals and diseases (data from CTD).

The Cancer Therapeutics Response Portal (CTRP) was used to link genetic, lineage and other cellular features of cancer cell lines to small-molecule sensitivity and Genomics of Drug Sensitivity in Cancer (GDSC) portal was used to link drug sensitivity in cancer cells and molecular markers of drug response. We found that NUDCD1 expression was significantly and negatively correlated with the CTRP drug sensitivity (IC_50_) for BRD-K01737880, BRD-K33514849 and GSK-J4 (Figure 8A and Supplementary Table 1). However, NUDCD1 expression was significantly and positively related to GDSC IC_50_ for PLX4720, SB590885 and Selumetinib while significantly and negatively correlated with NPK76-II-72-1, AICAR and BX-795 (Figure 8B and Supplementary Table 2). The CTD was also used to establish an interaction network between NUDCD1-chemicals-cancers (Figure 8C).

### Enrichment analysis of NUDCD1-related partners

**Figure 9:**
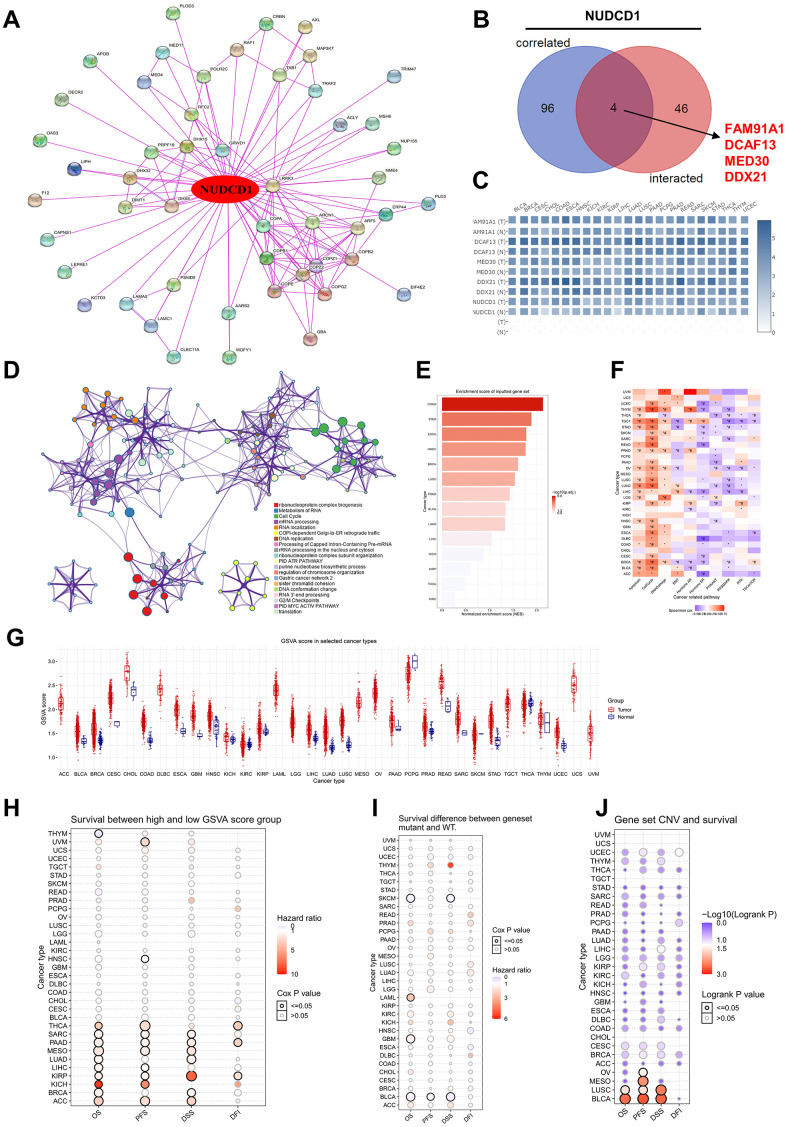
**Enrichment analysis of NUDCD1-related partners.** (**A**) NUDCD1-interacting proteins identified using STRING. (**B**) Wayne diagrams of intersection analyses of NUDCD1-correlated and inter-acting genes (data from GEPIA2 and STRING). (**C**) Corresponding heat maps of 4 NUDCD1-related genes in specific cancer types (data from GEPIA2). (**D**) Pathway and process enrichment analysis of 146 NUDCD1-related genes; a subset of enriched terms was selected and rendered as a network plot as indicated. (**E**) Enrichment scores for NUDCD1-related genes in the detailed cancers (data from GSCA). (**F**) Associations between GSVA score and activity of cancer related pathways (data from GSCA). (**G**) GSVA scores of NUDCD1-related genes in the detailed cancer types. (**H**) Survival differences between high and low GSVA score groups in multiple cancers (data from GSCA). (**I**) Survival differences between NUDCD1-related gene set mutant and WT (data from GSCA). (**J**) Survival differences between NUDCD1-related gene set CNV groups (amplification, deletion and WT) from GSCA database.

To further explore the molecular mechanism of NUDCD1 in human cancers, we established a protein-protein interaction (PPI) network including 50 NUDCD1-interacted proteins derived from STRING (Figure 9A). The top 100 genes that correlated with NUDCD1 expression indicated 4 common members: FAM91A1, DCAF13, MED30 and DDX21 (Figure 9B), and their corresponding expression in different tumor types could be established (Figure 9C). 146 NUDCD1-related -interacting and -correlated genes were also identified (Figure 9D). Protein-protein interactions were also identified for NUDCD1-related genes (Supplementary Figure 11A) and included 6 MCODE algorithm components (Supplementary Figure 11B). NUDCD1-related genes were compiled according to gene set enrichment analysis (GSEA) and gene set variable analysis (GSVA) scores to investigate genomic variations and clinical outcomes. The highest GSEA scores were found for COAD, STAD and ESCA (Figure 9E and Supplementary Figure 11C). NUDCD1-related genes were correlated with the cancer-related pathways of apoptosis, cell cycle and DNA damage (Figure 9F).

The GSVA scores in different tumors and subtypes of cancers are summarized in Figure 9G and Supplementary Figure 12A, respectively. Most cancer types when compared to non-tumor tissues possessed significantly higher GSVA scores in tumors. These scores were also linked to clinical/mutation/CNV/expression in THYM, UVM, HNSC, THCA, SARC, PAAD, MESO (Mesothelioma), LUAD, LIHC, KIRP, KICH, BRCA and ACC (Adrenocortical carcinoma). The high GSVA score groups had a higher hazard ratio for survival (Figure 9H). THYM, SKCM (Skin Cutaneous Melanoma), LAML, GBM, BLCA patients with mutation of NUDCD1-related gene set suggests shorter survival than those in the WT group (Figure 9I). There was an association identified between the NUDCD1-related gene set and CNV survival (Figure 9J). The NUDCD1-related gene set was also negatively correlated with immune infiltration score, CD4-T, NK, Tfh, MAIT, NKT, Gamma delta, CD8-T, cytotoxic and Th2; while positively correlated with nTreg, neutrophil, central memory and iTreg (Supplementary Figure 12B).

The association between NUDCD1-related gene set mutation and immune infiltration in different cancer types is summarized in Supplementary Figure 12C. NUDCD1-related genes also impacted drug susceptibility. The expression of NUDCD1-related gene set was negatively correlated CTRP and GDSC drug susceptibility (IC_50_) in most cases. Notably, expression of NUDCD1-related gene set (TXNRDI, STK3, PSMD5, DHX32, COPZ2 and COPB2) was positively correlated with the drug susceptibility (IC_50_) in both CTRP (Supplementary Figure 12D) and GDSC (Supplementary Figure 12E).

### Validation of NUDCD1 expression and function in STAD

**Figure 10:**
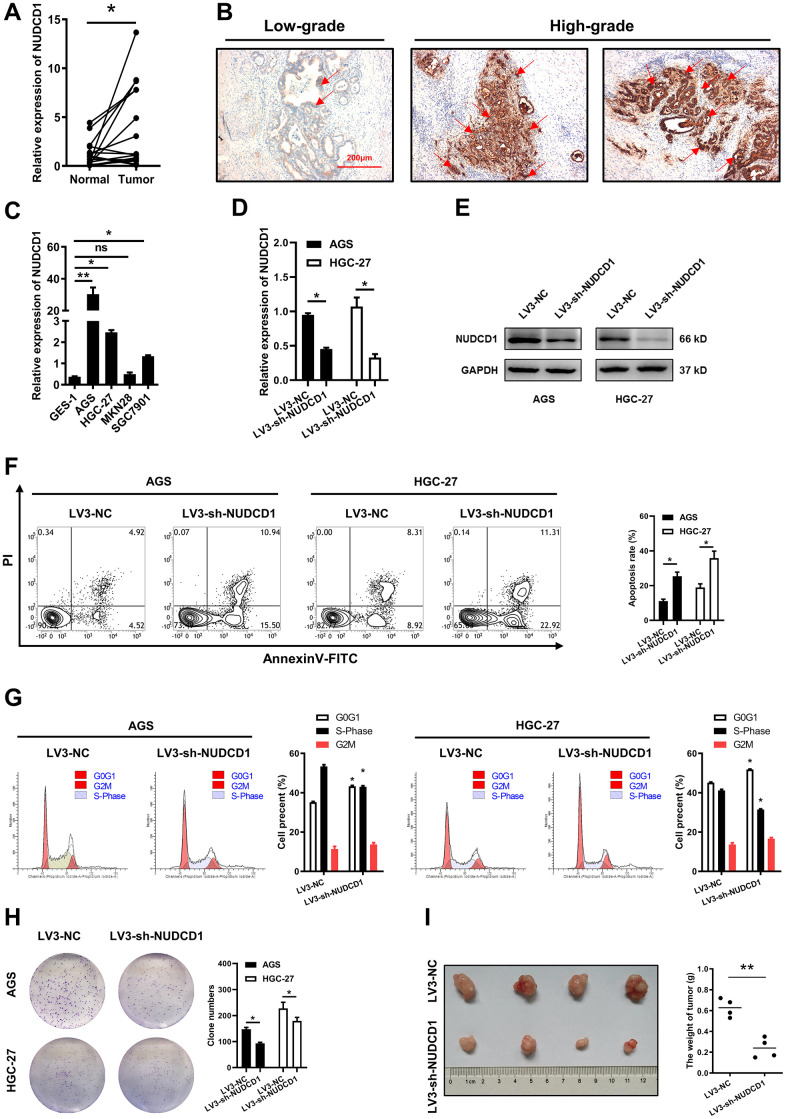
**Validation of NUDCD1 expression and function in STAD.** (**A**) Relative NUDCD1 expression in STAD tissues and adjacent pericarcinous tissues. (**B**) Immunohistochemistry analysis of NUDCD1 expression in STAD tissues. (**C**) Relative NUDCD1 expression in the normal gastric mucosal cell line, GES-1, and four STAD cell lines, AGS, HGC-27, MKN28 and SGC7901. (**D**) Relative mRNA and (**E**) protein expression of NUDCD1 in AGS and HGC-27 cells with LV3-NC or LV3-sh-NUDCD1. (**F**) The ratio of apoptosis in AGS and HGC-27 cells with LV3-NC or LV3-sh-NUDCD1. (**G**) Cell cycle in AGS and HGC-27 cells with LV3-NC or LV3-sh-NUDCD1. (**H**) Colony formation assay results in AGS and HGC-27 cells with LV3-NC or LV3-sh-NUDCD1. (**I**) Weight of subcutaneously xenografted STAD in nude mice.

To further validate our results, we examined NUDCD1 mRNA and protein expression using STAD and pericarcinous tissues. We found that the mRNA expression of NUDCD1 was significantly higher than that in pericarcinous tissues (Figure 10A); moderate and strong staining of NUDCD1 was mainly observed in high-grade STAD tissues, while most of the low-grade tumor tissues showed weak staining (Figure 10B). Among the five human cell lines (CES-1, AGS, SGC7901, MKN28 and HGC-27), NUDCD1 showed highest expression in AGS and HGC-27 (Figure 10C). Next, stably NUDCD1-knockdown or NC AGS and HGC-27 cells were constructed and validated (Figure 10D, 10E). Based on above, NUDCD1 was correlated with the cancer-related pathways of apoptosis and cell cycle in STAD (Figure 9F), so we detected the apoptosis and cycle in AGS and HGC-27 cells. Results showed that NUDCD1-knockdown increased the percentage of apoptotic STAD cells (Figure 10F); moreover, elevated the proportion of cells in G0/G1 phase and decreased proportion of cells in S phase were also observed in the comparison to negative control (Figure 10G). As expected, colony formation assays revealed that NUDCD1 knockdown significantly decreased the formation capacity *in vitro* (Figure 10H). Consistent with that, to knockdown NUDCD1 *in vivo* could significantly suppress the carcinogenesis of STAD cells in nude mice (Figure 10I).

## DISCUSSION

NUDCD1 was originally identified in a chronic myelogenous leukemia cDNA expression library and is a highly immunogenic protein [18]. NUDCD1 is also multifunctional and involved in the regulation of cellular biological processes especially in tumor cells [19–21]. As an oncoprotein upregulated in multiple tumor tissues and cell lines, NUDCD1 has been reported to contribute to ovarian and cervical cancer [16, 18], colorectal cancer [12], and renal cell carcinoma [20, 22]. We found that NUDCD1 was widely expressed in human tissues including immune cells and especially CD8+ and CD4+ T cells. NUDCD1 overexpression has been documented for NSCLC [15], colorectal cancer (CRC) [12], renal carcinoma [20] and ovarian and cervical cancer [16]. Additionally, NUDCD1 overexpression was also associated with poor survival of patients with hepatocellular carcinoma [23] and head and neck squamous cell carcinoma [24]. Consistent with these reports, we found NUDCD1 was predominantly expressed in a variety of tumors compared with normal tissues. In some tumor types, it was associated with cancer grade, subtype and stage. We verified that high NUDCD1 expression was closely related with poor prognoses in BRCA, LUAD and SARC. An independent prognostic analysis of NUDCD1 from clinical bioinformatics (ACLBI) data showed that NUDCD1 expression was a moderate risk in the clinical prognosis of 1-year and 5-year survival for SARC. Whether NUDCD1 functions as a biomarker for cancers still requires investigation.

We then tested the genetic and epigenetic features of NUDCD1 in cancers. In particular, missense mutations can render the resulting protein nonfunctional and this was the primary type of mutation we found for NUDCD1 and could provide a growth advantage and perhaps metastatic potential [25]. Notably, the mutation frequencies of NUDCD1 appeared to be higher in some cancers such as UCEC and LUAD. Mutation of NUDCD1 may influence the DFS and PFS in cancer patients and SNV patients had a longer PFS versus NUDCD1-WT patients in UCEC. Moreover, the CNV patients had significantly worse DFI, DSS, OS and PFS in KIRP and UCEC. In addition, we found that NUDCD1 methylation was significantly negatively correlated with its expression in most cancer types. These results demonstrated that the dysregulated expression of NUDCD1 may be partially mediated by DNA methylation. As one of the most common epigenetic modifications in mammals, methylation causes inactivation of certain tumor-suppressor genes and contributes to cell transformation [26]. In UVM, KICH and SARC, NUDCD1 methylation may prolong survival. In conjunction with these findings, SARC was the only cancer type which survival was influenced by both the expression and methylation of NUDCD1. CNV of NUDCD1 which was observed in various tumors could be involved in the alteration of gene transcription, DNA methylation, mRNA stability or aberrant transcriptional factor, etc. Removal of these factors which could attenuate the NUDCD1 level might be a potential strategy for tumor therapy, so it is hopeful to explore the underlying mechanism. Overall, determining the genetic and epigenetic features of NUDCD1 expression may assist in tailoring therapy to individual patient tumors.

Structurally, NUDCD1 may play a key role in immunity since we found a high level of expression in CML and this may provide a clue to its immune function. The tumor immune microenvironment consists of cancer cells, blood vessels and immune infiltrates and this complex milieu provides the functional space for NUDCD1 in a contact or non-contact manner. In clinical experiments, the SEREX-identified tumor antigen CML66L elicits T cell immune responses [27] and antigen CML66 expression may play a role in shaping the post- donor lymphocyte infusion antibody repertoire [28]. However, whether NUDCD1 directly affects the immune system is still inconclusive. We found correlations between NUDCD1 expression and TILs, immunoinhibitors, immunostimulators, MHC, chemokines and their receptors. NUDCD1 expression was positively related to ACT CD4, TH2, CD274, CXCL8, CXCL10, CXCL11 while negatively related to monocyte, LGAL59, TNFRSF14, TAPBP, CCL14 and CCR10. More importantly, NUDCD1 possessed significant mutation differences between responders and non-responders of ipilimumab therapy in melanoma patents suggesting that the therapeutic effect of this mAb may be predicted by NUDCD1 mutations. DNA mismatch repair deficiency and subsequent microsatellite instability (MSI), hypermutator phenotype secondary to frequent polymorphism in short repetitive DNA sequences and single nucleotide substitution [29] lead to the accumulation of mutation loads in cancer-related genes and the aggravation of tumor mutation burden (TMB) are responsible for tumor initiation [30]. Herein, we found correlations between NUDCD1 expression and MSI and TMB across tumor types. Another key finding of this study was that NUDCD1 was associated with immune infiltration. The tumor related-immune microenvironment has significant implications for cancer progression. For instance, M2 tumor-associated macrophages stimulate tumor angiogenesis and contribute to immunosuppressive tumor microenvironment [31] and colorectal cancer metastasis could be promoted by AGR2 from tumor-associated neutrophils [32]. The frequency of Tumor specific CD8+ T cells can now be increased in cancer patients and enhancing tumor T cell infiltration is one way to improve cancer immunotherapy [33]. NUDCD1 expression was associated with the abundance of immune infiltrates especially CD4+, CD8+, macrophages, neutrophils, CAP, common myeloid progenitor X cell and MDSC. In the cancers with higher mutation frequencies, the NUDCD1 mutation was also linked to immune infiltration in UCEC. Exhausted, Th1, effector memory and DC infiltrates were significantly increased in NUDCD1-mutation patients in comparison to NUDCD1-WT patients although MAIT and Th17 infiltrates were significantly decreased. The methylation and CNV of NUDCD1 could also affect immune cell infiltrations. Thus, as a tumor-related antigen, NUDCD1 may play multiple roles in different tumor types and could impact tumor immunity.

Oncogenes are sometimes also associated with drug resistance or susceptibility. For instance, pleiotrophin affects the susceptibility of prostate cancer cells to cisplatin [34]. The mechanism of resistance to drugs is typically the result of alterations in tumor cell phenotype [35] and chemical-based modelling has been widely applied in cancer cell biology [36]. NUDCD1 was able to induce the oncogenic transformation and develop greater resistance to 5-fluorouracil-induced apoptosis in NIH 3T3 cells [21]. In the current study, NUDCD1 expression was negatively correlated CTRP and GDSC drug susceptibility in most cancers. As similar as the existing literature [12, 21], a positive correlation was observed between the expression of NUDCD1 mRNA and the IC50 of MEK inhibitors (Trametinib, Selumetinib) and PI3K inhibitor (TGX221). Otherwise, our analyses demonstrated that NUDCD1 weakened the effect of B-Raf inhibition (SB-590885, PLX-4720, Dabrafenib) and EGFR blocking (Cetuximab, Gefitinib), which to the best of our knowledge has not been previously reported. Our future investigations will validate these findings and elucidate the underlying mechanisms. Notably, a network of NUDCD1-chemicals-cancers established in our study may provide an alternative insight in cancer drug screening applications.

In CRC and PC cells, NUDCD1 can induce a complex-integrated phenotype to affect cell proliferation, migration, invasion and apoptosis via the EMT [12, 13]. Moreover, NUDCD1 overexpression in cancer cell lines promoted VEGF secretion, tumor growth and angiogenesis *in vitro* and *in vivo* [16]. Conversely, NUDCD1 silencing inhibited HeLa cell proliferation, metastasis and invasion [18]. Hence, in order to further explore the mechanism whereby NUDCD1 influences tumor progress, we integrated the co-expression network and analysis with the NUDCD1-related gene set. We screened the NUDCD1-interacted proteins from STRING and NUDCD1-correlated proteins from GEPIA2. The PPI and GSCA results were combined to establish a GSEA enrichment analysis that indicated that NUDCD1 can affect multiple tumors signaling pathways. For example, NUDCD1 promotes tumor angiogenesis and progression through enhancing autocrine VEGF-VEGFR2 signaling [16] and promotes the proliferation and metastasis of non-small cell lung cancer cells through the IGF1R-ERK1/2 activation [15]. We previously demonstrated that NUDCD1 expression is increased in CRC tissues while its silencing inhibits CRC cell EMT and arrested the cell cycle and increased apoptosis. Consistent with our previous research, NUDCD1-related genes were positively correlated with the pathways of apoptosis, cell cycle and DNA damage in most cancer types. Notably, cell cycle was an important gene cluster module in the PPI network although the regulatory mechanisms await supporting experimental data. NUDCD1-related genes were observed to regulate survival, immune infiltration and drug susceptibility and the NUDCD1-related gene set was positively correlated with the drug susceptibility of TXNRDI, STK3, PSMD5, DHX32, COPZ2 and COPB2 in both CTRP and GDSC. DHX15 was shown to interact physically with the first isoform of NUDCD1 [11] and may have sufficient value for further investigation. Lastly, based on the existing literature and analysis in our study, the laboratory verification of NUDCD1 role in STAD was conducted with our clinical samples and cancer cells.

## CONCLUSIONS

Together, these analyses using multiple databases confirmed the expression pattern of NUDCD1 across many cancer types. Although the prognostic value of NUDCD1 across pan-cancers was weak, the expression of NUDCD1 still significantly affected the survival of many cancers. Additionally, we analyzed genetic and epigenetic features of NUDCD1 expression and a deeper analysis revealed the role of NUDCD1 in the tumor-immune system and tumor-immune infiltration as an antigen. For the first time, a complete enrichment analysis of NUDCD1-related genes showed an integrated network for the clinical/epigenetic features /immune infiltration/pathways/ drug susceptibility.

## MATERIALS AND METHODS

### Expression analysis of NUDCD1

The mRNA expression patterns of NUDCD1 in normal tissues from the Human Protein Atlas (HPA) project (http://www.proteinatlas.org) [37] derived from RNA-seq data was obtained. NUDCD1 protein expression has been identified for each of the 44 human tissues and the immunohistochemistry data of NUDCD1 in normal and cancer tissues was obtained from the “Human pathology” module of HPA. The bulk tissue gene expression and single tissue expression for NUDCD1 was obtained from the Genotype-Tissue Expression (GTEx) project (http://www.gtexportal.org) [38].

Differences in NUDCD1 expression between tumor and adjacent normal tissues in multiple cancers was obtained from the “Gene_DE” module of TIMER2 (Tumor Immune Estimation Resource, Version 2) web (http://timer.cistrome.org/) [39]. For the tumor entries that did not contain normal tissue counterparts, we drew box plots of NUDCD1 expression in tumor and adjacent normal tissues from the “Expression DIY” module of GEPIA2 (Gene Expression Profiling Interactive Analysis, Version 2) server (http://gepia2.cancer-pku.cn/) [40] using the settings |Log_2_FC| cutoff =1, P-value cutoff = 0.01 and “Match TCGA normal and GTEx data”. For BRCA (breast invasive carcinoma), NUDCD1 expression in different tumor subtypes was obtained from TISIDB (web portal for tumor and immune system interaction) web (http://cis.hku.hk/TISIDB/index.php) [41].

Protein expression analysis of NUDCD1 was conducted using the CPTAC (Clinical Proteomic Tumor Analysis Consortium) dataset in UALCAN (The University of ALabama at Birmingham CANcer data analysis Portal) portal (http://ualcan.path.uab.edu/analysis-prot.html) [42]. From Gene Set Cancer Analysis (GSCA) platform (http://bioinfo.life.hust.edu.cn/GSCA/#/) [43], gene enrichment across pan-cancers was based on gene set enrichment analysis (GSEA) scores of NUDCD1 and associations between pathway activity and NUDCD1 expression scores were compiled using gene set variation analysis (GSVA).

### Survival analysis

The “clinical” module of TISIDB was used to obtain associations between NUDCD1 expression and overall survival across human cancer types. The “survival analysis” module of GEPIA2 was used to conduct the overall survival analysis of NUDCD1 across all TCGA tumors using the settings cutoff-high (50%) and cutoff-low (50%) values as thresholds. The KM (Kaplan-Meier) Plotter http://kmplot.com/analysis/index.php?p=background) [44] for pan-cancer was used to correlate NUDCD1 expression and overall survival in 21 tumor types under the auto best cutoff value. Survival differences including disease-free interval (DFI), disease-specific survival (DSS), overall survival (OS) and progression-free survival (PFS) between high and low NUDCD1 expression groups was revealed using GSCA. The combined outcomes from these 4 datasets was used to identify tumor types in which NUDCD1 possessed a potential prognostic value and were summarized using the Venn web tool Calculate (http://bioinformatics.psb.ugent.be/webtools/Venn/).

### Genetic and epigenetic alteration analysis

The cBioPortal (cBio Cancer Genomics Portal, http://www.cbioportal.org) [45] was employed to analyze genetic and epigenetic alterations in NUDCD1. The alteration frequency of NUDCD1 across different tumors was summarized in the “Cancer types summary” module. Using the “Plots” module, general mutation counts of NUDCD1 in various TCGA cancer types were then described. For cancer patients possessing mutant or wild type (WT) NUDCD1, the DFS (disease-free survival) and PFS were examined using the “survival” module. The mutation types (e.g., missense, truncating and splice) and mutated site information in detailed cancers were annotated by the “mutations” module. In addition, the SNV (Single Nucleotide Variation) percentage of NUDCD1 and CNV (Copy Number Variation) percentage of NUDCD1 in different cancers was provided by the “mutations” module of GSCA. The “mutations” module of GSCA also assessed the survival differences between cancer patients with NUDCD1 mutant and WT signatures and was used to display correlations between CNV and survival across different tumors.

### Methylation of NUDCD1 in cancers

Methylation differences between tumor and normal samples of NUDCD1 across different cancer types from TCGA was analyzed by the “mutations” module of GSCA using the thresholds >10 pairs of tumor-normal samples and p-value≤0.05. The GSCA web server was also used to summarize correlation profiles between methylation and NUDCD1 mRNA expression in specific cancers. The “mutations” module of GSCA was used to analyze survival differences (DFI, DSS, OS, and PFS) between cancer patients with high and low methylation level of NUDCD1.

### Tumor-immune system and NUDCD1

We used the TISIDB dataset to analyze relationships between the abundance of tumor-infiltrating lymphocytes (TILs) and NUDCD1 expression with the “lymphocyte” module; and relationships between 3 immunomodulator types (inhibitor, stimulator and MHC molecule) and NUDCD1 were examined using the “immunomodulator” module; correlations between chemokines (or receptors) and NUDCD1 levels were assessed with the “chemokine” module. We also used TISIDB to assess whether NUDCD1 had a significant expression or mutational difference between responders and non-responders to immunotherapy (e.g., PD-1, PD-L1, CTLA-4).

In this study, “pan-cancer” module of assistant for clinical bioinformatics (ACLBI) tool (https://www.aclbi.com) was employed to investigate the subsequent microsatellite instability (MSI) and aggravation of tumor mutation burden (TMB) in various human cancers.

### Immune infiltration in cancer

The “Gene module” in TIMER2 allowed us to visualize correlations between NUDCD1 expression with immune infiltration in numerous and diverse cancer types. The NUDCD1 and immune infiltrates information was converted to heatmaps to show the purity-adjusted Spearman's rho coefficients across various cancer types. We also assessed correlations of NUDCD1 with markers for immune cell subsets including CD8+ T cells, total T cells, B cells, monocytes, tumor-associated macrophages (TAMs), M1 and M2 macrophages, neutrophils, NK cells, DCs, Th1 cells type 2 helper T cell (Th2), Tfh cells, type 17 helper T cell (Th17), Tregs and exhausted T cells.

### Drug sensitivity

In the “Drug sensitivity” module of GSCA, there were two sources of data: Cancer Therapeutics Response Portal (CTRP) [46] and Genomics of Drug Sensitivity in Cancer (GDSC) [47]. CTRP contains 481 small molecules with IC_50_, 1001 cell lines and 18900 genes while GDSC includes 265 small molecules, 860 cell lines and 17419 genes. We explored correlations between NUDCD1 expression and drug IC_50_ (the half maximal inhibitory concentration).

The Comparative Toxicogenomics Database (CTD, http://ctdbase.org/) [48] provides manually curated information about chemical-gene/protein interactions as well as chemical-disease and gene-disease relationships. These data are integrated with functional and pathway data to aid in development of hypotheses concerning mechanisms underlying environmentally influenced diseases. We downloaded the data “Chemical-gene interactions” and “Gene-disease associations” to sort the NUDCD1 related chemicals and disease and then established an interaction network of NUDCD1-chemicals-cancers.

### Enrichment analysis of NUDCD1-related partners

We input NUDCD1 in the “search single protein by name” module of STRING (search tool for the retrieval of interacting genes/proteins) website (https://string-db.org/) [49] to obtain NUDCD1-interacted proteins. The primary parameters were set as follows: organism “homo sapiens”, meaning of network edges (“evidence”), active interaction sources (“experiments” and “co-expression”), minimum required interaction score [“Low confidence (0.150)”] and max number of interactors to show (“no more than 50 interactors” in 1st shell). NUDCD1-correlated targeting genes in TCGA tumor and TCGA normal tissues were obtained from the “Similar Gene Detection” module of GEPIA2, the parameter “Top # similar Genes” was set as “100”. The genes encoding NUDCD1-interacting proteins and NUDCD1-correlated targets were then listed and the Venn diagram viewer was used to screen out coincident genes. In the “Multiple Gene Comparison” pane in the “Expression DIY” module of GEPIA2, we profiled tissue-wise expression of coincident genes in TCGA cancer and matched normal tissues. NUDCD1-interacting and -correlated genes were identified using the pathway and process enrichment analysis in METASCAPE (https://metascape.org/gp) [50]. We obtained GSEA and GSVA scores of NUDCD1-related (-interacting and -correlated) genes from GSCA. Enrichment of NUDCD1-related genes across pan-cancers were performed based on the GSEA score. Based on the clinical/mutation/CNV/expression of the NUDCD1-related gene set, the GSVA score was compiled and we explored associations between pathway activity and expression score of NUDCD1-related genes and then compared the GSVA score of NUDCD1-related genes in different cancers. The relationships between GSVA score and survival, survival differences between gene set mutant and wild type and the corrections between gene set CNV and survival were all systematically assessed by GSCA.

### The role of NUDCD1 in STAD samples and cells

To the best of our knowledge, the expression and function of NUDCD1 in STAD tissues and cells was rarely reported. So, we detected the expression of NUDCD1 in normal and cancer tissues from 16 STAD patients by using quantitative real-time PCR and immunohistochemistry. Next, we constructed stably NUDCD1-knockdown or NC STAD cells using lentiviral vectors and analyzed the cell apoptosis or cycle using flow cytometry. Furthermore, the role of NUDCD1 in cell proliferation was determined by using colony formation *in vitro* and tumor xenograft *in vivo*. Detailed information and procedures for these molecular biological experiments are described in Supplementary Information. Human samples collection and all animal experiments were approved by the Ethics Review Board of Affiliated Hospital of North Sichuan Medical College.

### Statistical analysis

All data analysis were conducted using SPSS v20.0. Data are expressed as means ± standard errors of the mean (SEM). When data were normally distributed and had homogenous variances, the Student’s t-test was used for comparisons between two groups and one-way ANOVA followed by Dunnett’s post-hoc tests were used in comparisons between 3 or more groups; when the data violated the normality or homogeneity of variances, Mann-Whitney test followed by Tamhane's T2 test was performed in the comparisons between two groups and Kruskal-Wallis test followed by Dunnett's T3 tests was performed in the comparisons between 3 or more groups. *P* < 0.05 was considered statistically significant.

### Data availability statement

The original contributions presented in the study are included in the article, further inquiries can be directed to the corresponding author. A preprint has previously been published in the Research Square to the following links: https://www.researchsquare.com/article/rs-2225502/v1 and https://europepmc.org/article/ppr/ppr574609.

## Supplementary Material

Supplementary Information

Supplementary Figures

Supplementary Tables
