# Supplementary material for: Identifying the role of NUDCD1 in human tumors from clinical and molecular mechanisms: a study based on comprehensive bioinformatics and experimental validation
Source: Aging (Albany NY). 2023 Jun 19;15(12):5611–49. doi: 10.18632/aging.204813 (PMC10333089; doi:10.18632/aging.204813)
Supplement: Supplementary Information [file aging-15-204813-s001.pdf]

SUPPLEMENTARY INFORMATION

MATERIALS AND METHODS

Patient samples, immunohistochemistry (IHC) and ethical statements

16 cases of STAD patients who underwent radical surgery in the Department of Gastroenterology, Affiliated Hospital of North Sichuan Medical College, from Sep 2021 to Jan 2022, were included in the study. The STAD and normal pericarcinous tissues (parallel tissues ≥ 5 cm from the borders of cancerous tissues) were surgically resected. A part of each sample was quickly stored in liquid nitrogen for real-time PCR, and the rest was conducted to immunohistochemistry detection as described previously [1]. Samples collection was approved by the Ethics Review Board of Affiliated Hospital of North Sichuan Medical College.

Cell culture

Human gastric epithelium cell lines (GES-1 cells) and human GC cell lines (AGS, HGC-27, MKN28 and SGC-7901 cells) were purchased from ATCC. Cells were cultured with RPMI1640 medium (HyColony Logan, UT, USA) supplemented with 10% FBS (Gibco BRL) at 37° C in 5% CO2.

Plasmid construction and transfection

Lentiviral particles containing short hairpin RNA (shRNA) targeting NUDCD1 mRNA (termed LV3-sh-NUDCD1) or its negative control, a non-targeting RNA sequence (termed LV3-NC) were all designed, constructed, amplified, and purified by GenePharma (Shanghai, China). The NUDCD1-targeting shRNA sequence was 5'- GCTTGGAGATTTCCTTGATTA-3'. The control shRNA sequence was 5'-CAACAAGAT GAAGAGCACCAA-3'. AGS and HGC-27 cells were transduced with shRNA-expressing lentivirus as described previously [2].

RNA extraction, qRT-PCR and Western blot assays

RNA and protein extraction, qRT-PCR, and Western blot assays were performed as described previously [1]. The quality of nucleic acids was confirmed by gel electrophoresis. The mRNA and protein expression of NUDCD1 were normalized by the control GAPDH.

Cell apoptosis, cell cycle and cell proliferation *in vitro*

The apoptosis and cycle of AGS and HGC-27 cells (LV3-NC or LV3-sh-NUDCD1) were examined by flow cytometry as described previously [1]. For colony formation assays, AGS and HGC-27 cells were seeded at a density of 1000 cells per well in 24-well plates. After 10 days of growth, the cells were rinsed with PBS, fixed in 4% paraformaldehyde, and stained with 5% crystal violet for 30 min. Then, the colonies were characterized and counted for the evaluation of cell proliferation.

Cell proliferation *in vivo* and ethical statements

Female nude mice (5-6 weeks old BALB/c-nu/nu) were used for animal studies (purchased from the Beijing HFK Bioscience, Beijing, China). For *in vivo* tumor xenograft studies, 4×10^6 LV3-NC or LV3-sh-NUDCD1 AGS cells were re-suspended in 200 μL PBS and injected subcutaneously into the axillary fossae of female nude mice respectively (four mice per group) as described previously [1]. At 42 days post-injection, mice were sacrificed. Tumors were dissected and weighed. All animal experiments were approved by the Ethics Review Board of Affiliated Hospital of North Sichuan Medical College.

REFERENCES

1. Han B, Zhang YY, Xu K, Bai Y, Wan LH, Miao SK, Zhang KX, Zhang HW, Liu Y, Zhou LM. NUDCD1 promotes metastasis through inducing EMT and inhibiting apoptosis in colorectal cancer. Am J Cancer Res. 2018; 8:810–23. PMID:29888104

2. Teng YS, Chen WY, Yan ZB, Lv YP, Liu YG, Mao FY, Zhao YL, Peng LS, Cheng P, Duan MB, Chen W, Wang Y, Luo P, et al. L-Plastin Promotes Gastric Cancer Growth and Metastasis in a *Helicobacter pylori cagA*-ERK-SP1-Dependent Manner. Mol Cancer Res. 2021; 19:968–78. https://doi.org/10.1158/1541-7786.MCR-20-0936 PMID:33771880
