# Supplementary material for: Identifying the role of NUDCD1 in human tumors from clinical and molecular mechanisms: a study based on comprehensive bioinformatics and experimental validation
Source: Aging (Albany NY). 2023 Jun 19;15(12):5611–49. doi: 10.18632/aging.204813 (PMC10333089; doi:10.18632/aging.204813)
Supplement: Supplementary Figures [file aging-15-204813-s002.pdf]

SUPPLEMENTARY FIGURES

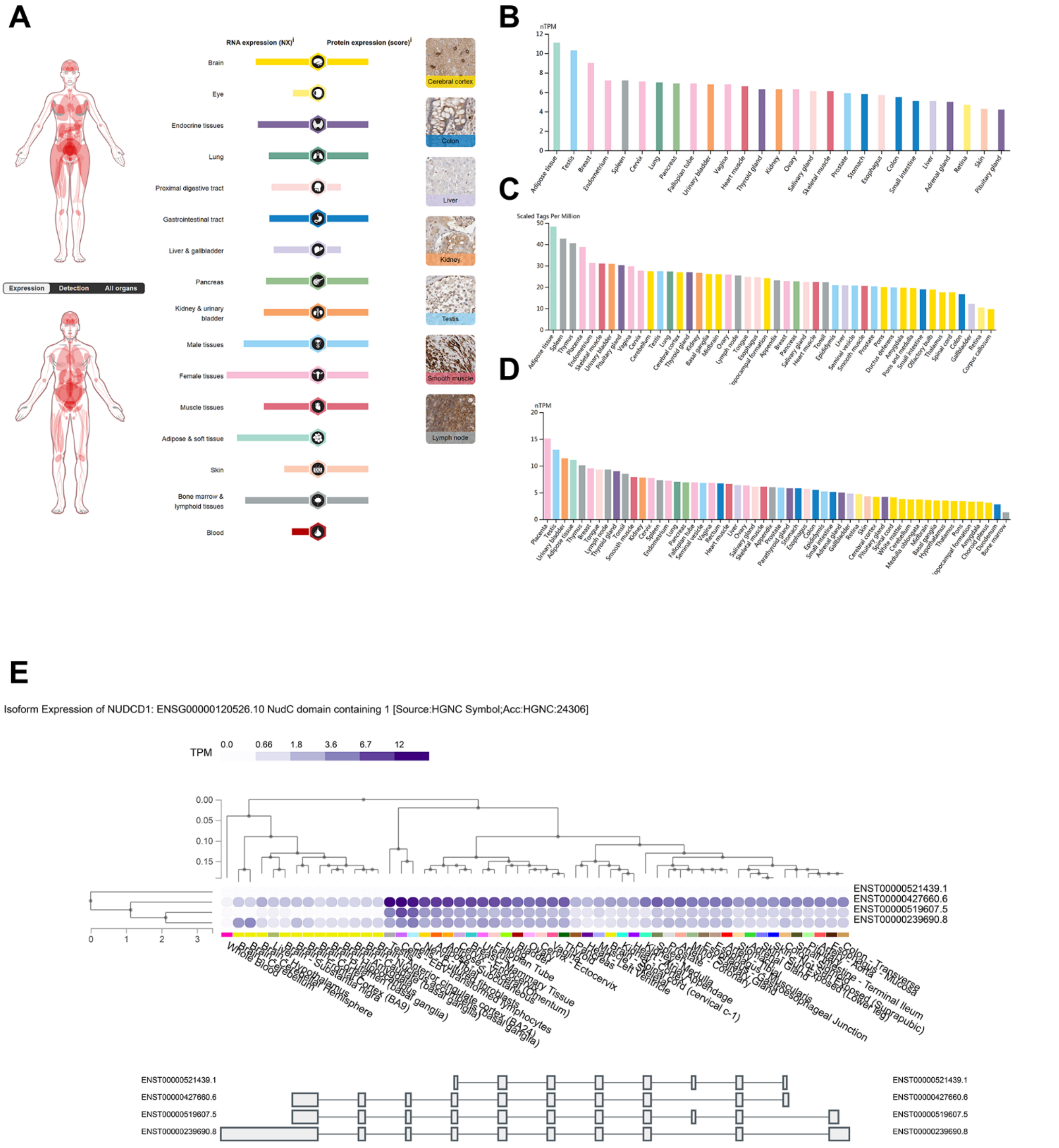

Supplementary Figure 1. The expression and Isoform of NUDCD1 in human tissues. (**A**) The RNA and protein expression summary of NUDCD1 in human tissues (data from HPA). (**B**) NUDCD1 expression levels in human tissues (data from GTEx). (**C**) NUDCD1 expression levels in human tissues (data from FANTOM5 dataset). (**D**) NUDCD1 expression levels in human tissues (data from consensus dataset). (**E**) Isoform expression of NUDCD1 (data from GTEx).

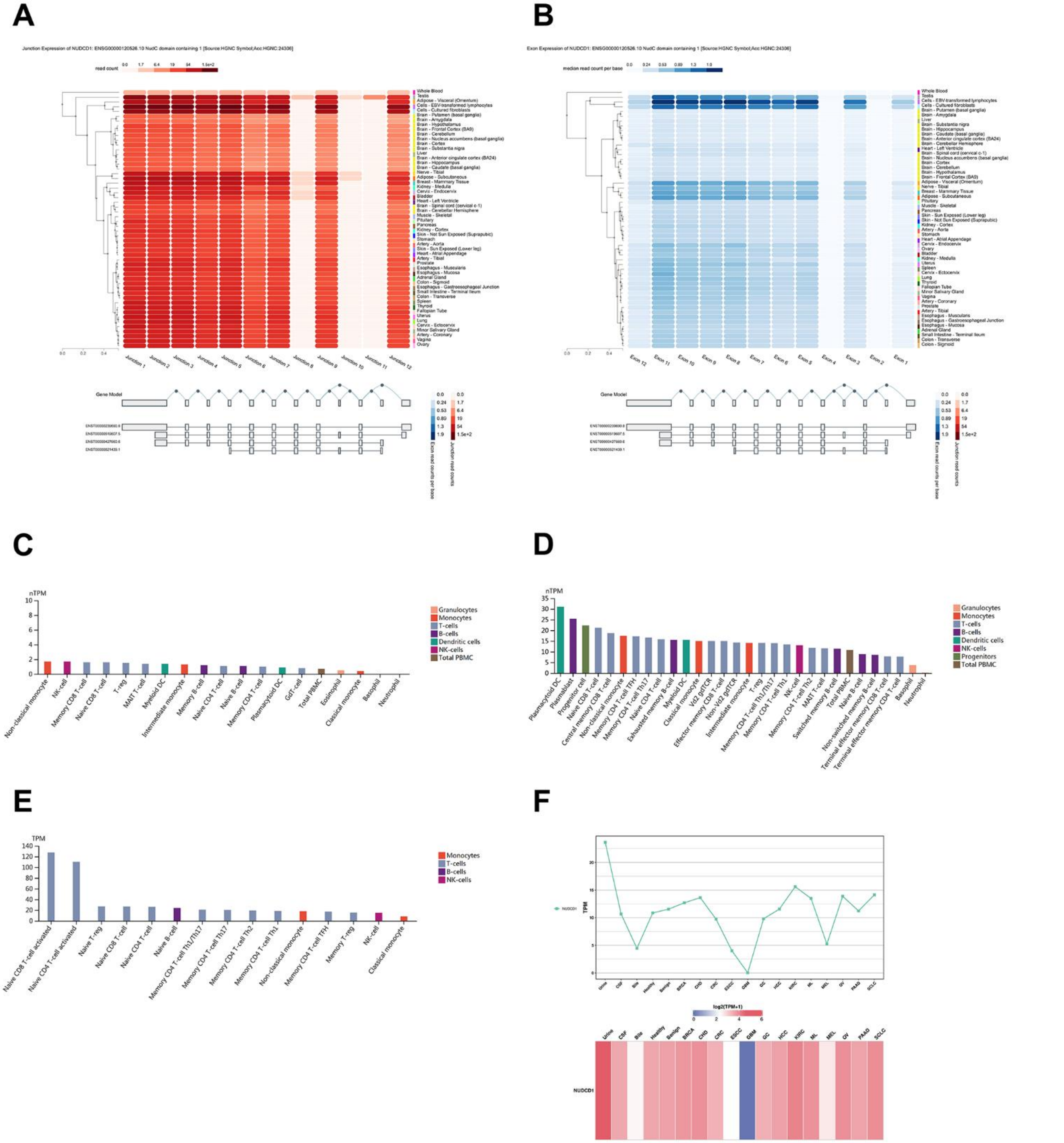

Supplementary Figure 2. The expression of NUDCD1 in junction, exon, blood cells and extracellular vesicles. Junction expression (**A**) and exon expression (**B**) of NUDCD1 (data from GTEx). (**C**) NUDCD1 expression levels in human blood cells (data from HPA). (**D**) NUDCD1 expression levels in human blood cells (data from Monaco scaled dataset). (**E**) NUDCD1 expression levels in human blood cells (data from Schmiedel dataset). (**F**) Line and heat map chart of NUDCD1 expression level in extracellular vesicles (data from exoRBase 2.0, http://www.exorbase.org/).

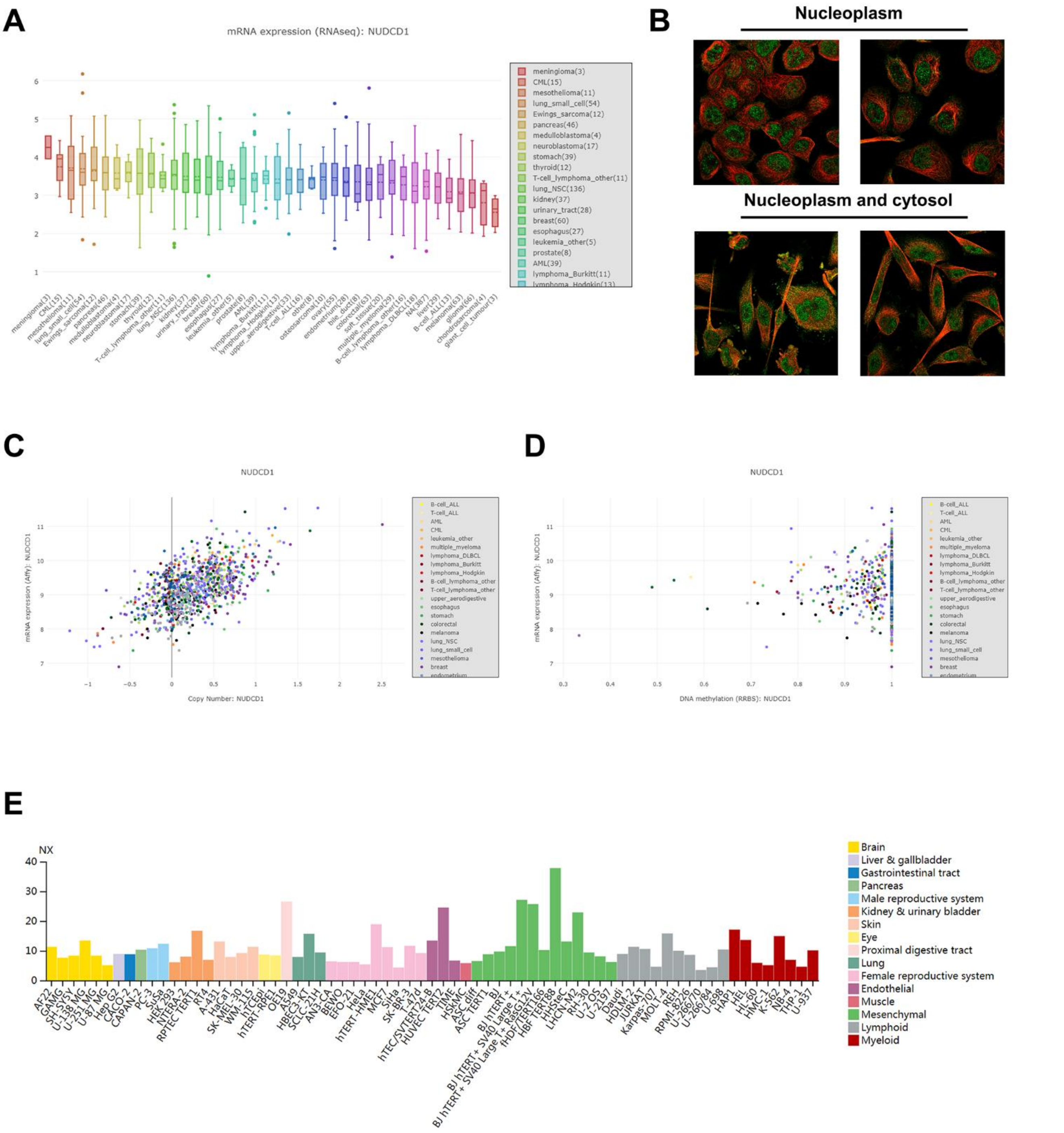

Supplementary Figure 3. NUDCD1 expression levels in different types of human cancer cells. (**A**) NUDCD1 mRNA expression across different cell lines from CCLE. (**B**) The subcellular location of NUDCD1 by indirect immunofluorescence microscopy, which was calculated by HPA. (**C**) Correlation between copy number variation and mRNA level of NUDCD1 across different cell lines (data from CCLE). (**D**) Correlation between promoter DNA methylation and mRNA level of NUDCD1 across different cell lines (data from CCLE). (**E**) Overview of NUDCD1 expression levels in different cell lines analyzed in the HPA.

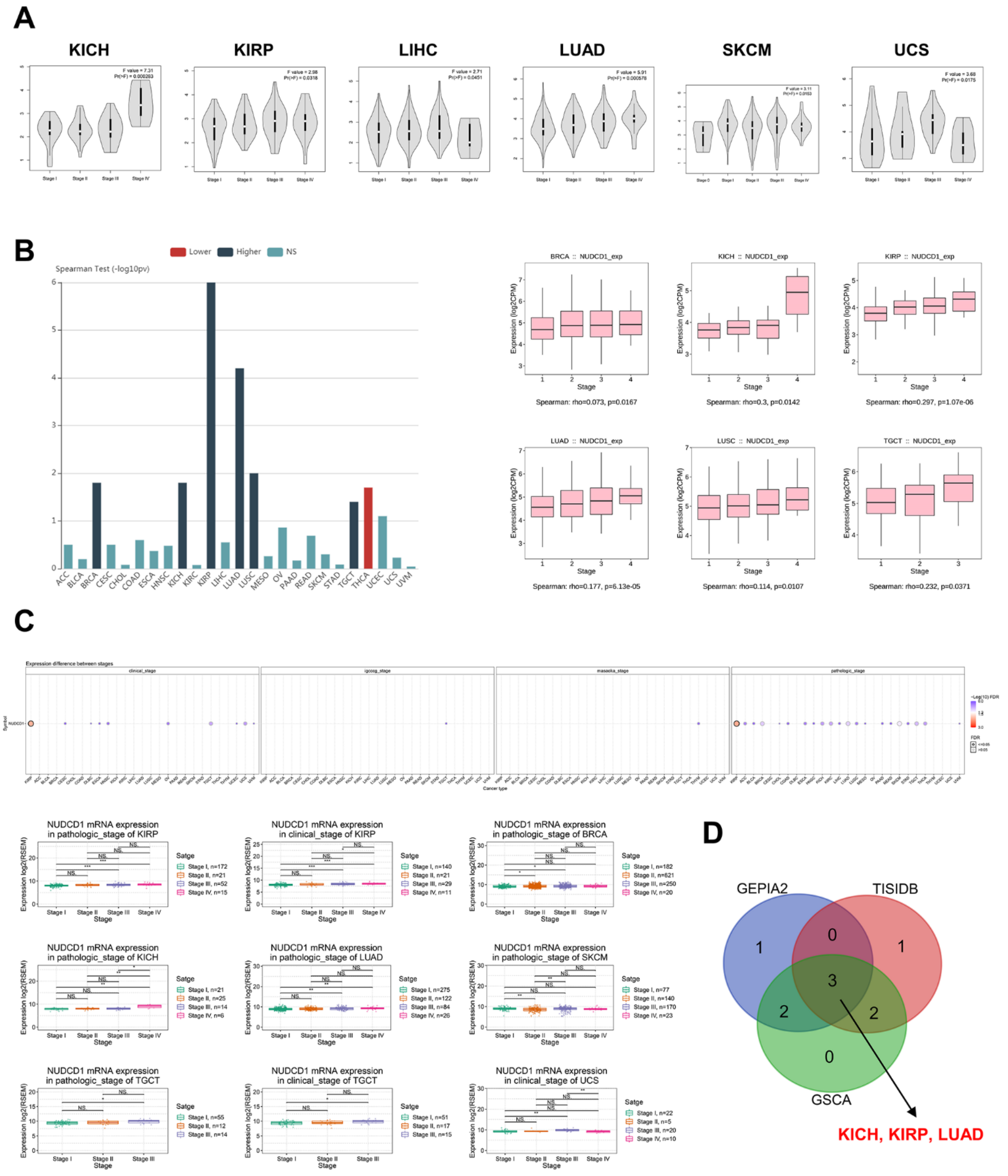

Supplementary Figure 4. Expression of NUDCD1 in different pathological stages of cancers. (**A**) Expression level of NUDCD1 in different pathological stages of KICH, KIRP, LIHC, LUAD, SKCM and UCS from GEPIA2. (**B**) Expression level of NUDCD1 in different pathological stages of multiple cancers from TISIDB. (**C**) The difference of NUDCD1 mRNA expression between stages in the specific cancers from GSCA. (**D**) Wayne diagram shows the cancers whose tumor stage associated with NUDCD1, data from GEPIA2, TISIDB and GSCA.

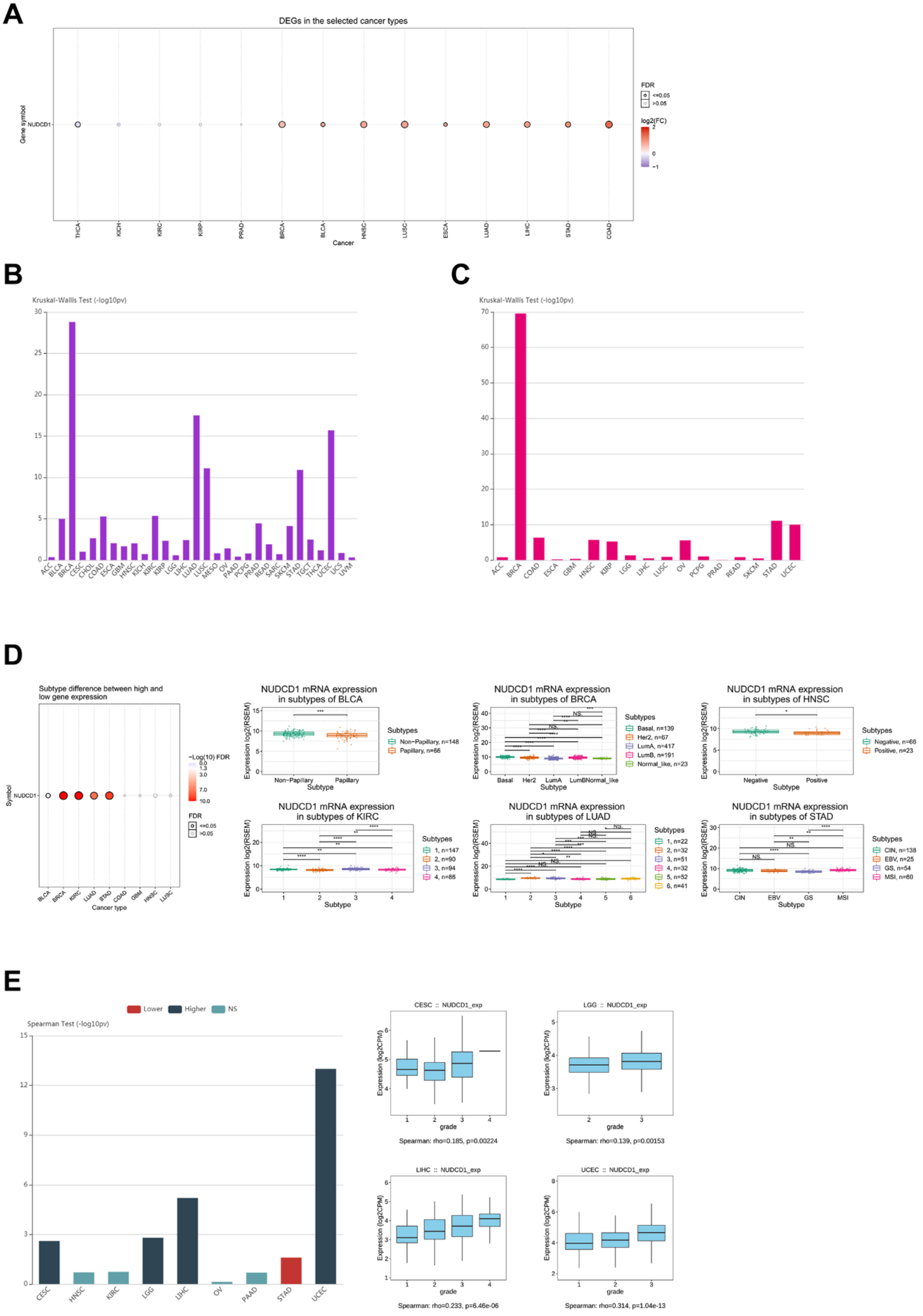

Supplementary Figure 5. NUDCD1 mRNA expression in different cancer subtypes and grades. (**A**) NUDCD1 mRNA expression between tumor and normal in multiple cancers (data from GSCA). Associations between NUDCD1 expression and immune subtypes (**B**), molecular subtypes (**C**) across human cancer (data from TISIDB). (**D**) Associations between subtypes and NUDCD1 expression across human cancers. (**E**) Associations between NUDCD1 expression and grades across human cancers.

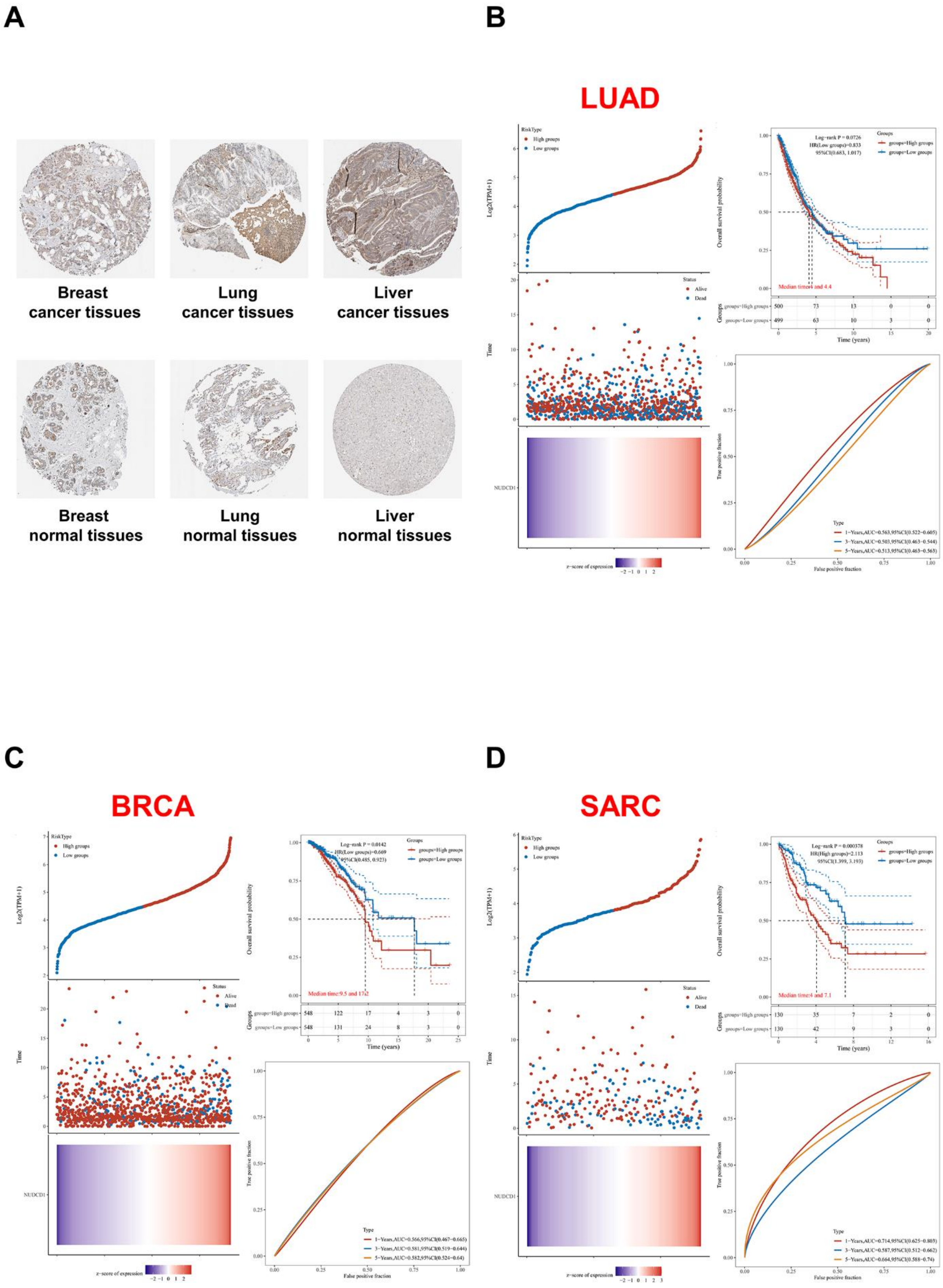

Supplementary Figure 6. The protein expression and prognostic analysis for NUDCD1 in LUAD, BRCA and SARC. (**A**) The IHC images of NUDCD1 in normal breast tissues, normal lung tissues, normal liver tissues, and breast cancer tissues, lung cancer tissues, liver cancer tissues. Prognostic analysis of NUDCD1 signature in LUAD (**B**), BRCA (**C**) and SARC (**D**) (data from https://www.aclbi.com).

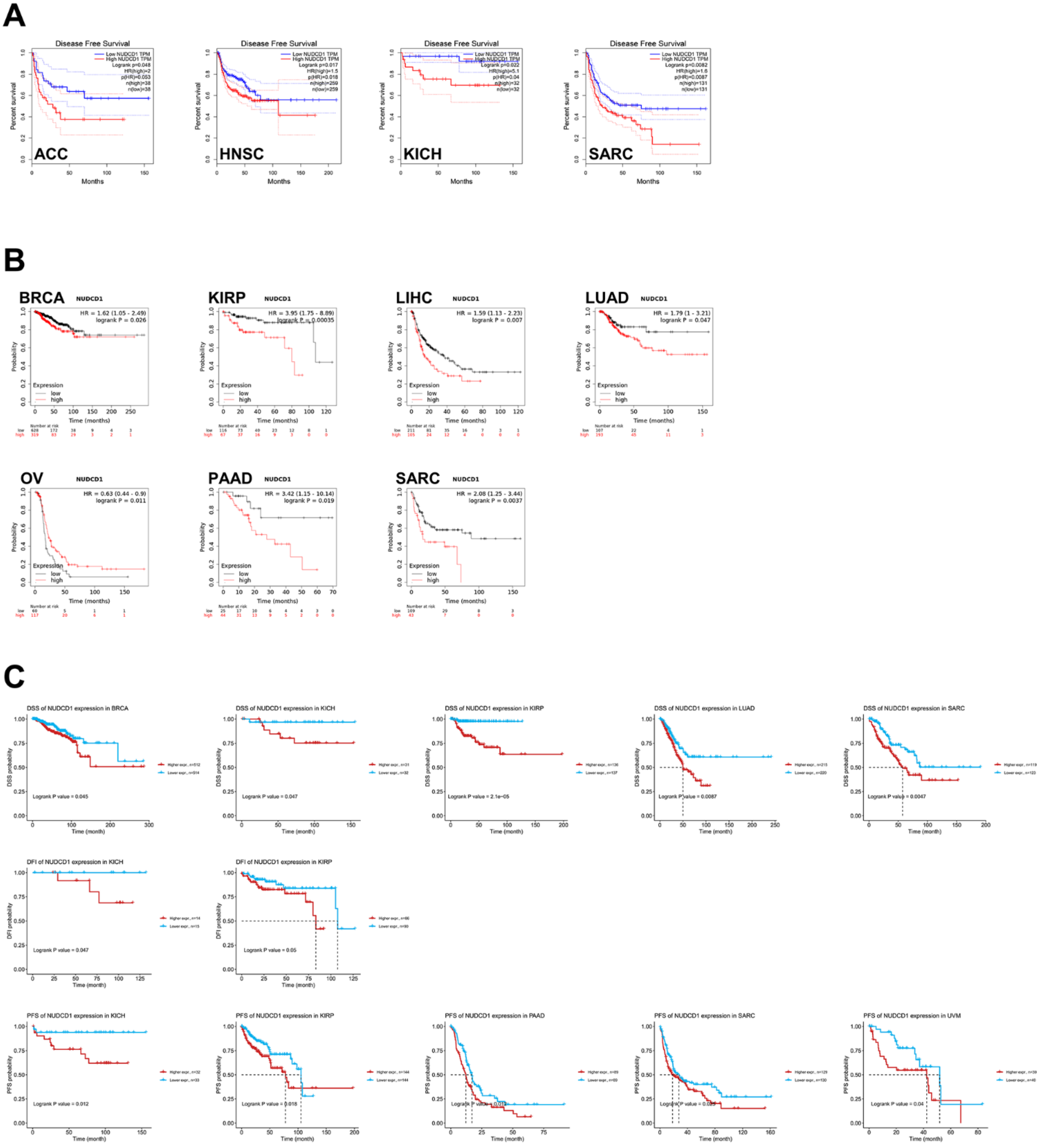

Supplementary Figure 7. Associations between NUDCD1 expression and DFS, RFS, DSS, DFI and PFS. (**A**) Associations between NUDCD1 expression and disease-free survival (DFS) in human cancers from GEPIA2. (**B**) Associations between NUDCD1 expression and relapse-free survival (RFS) in human cancers from KM plot. (**C**) DSS, DFI and PFS of NUDCD1 expression in human cancers (data from GSCA).

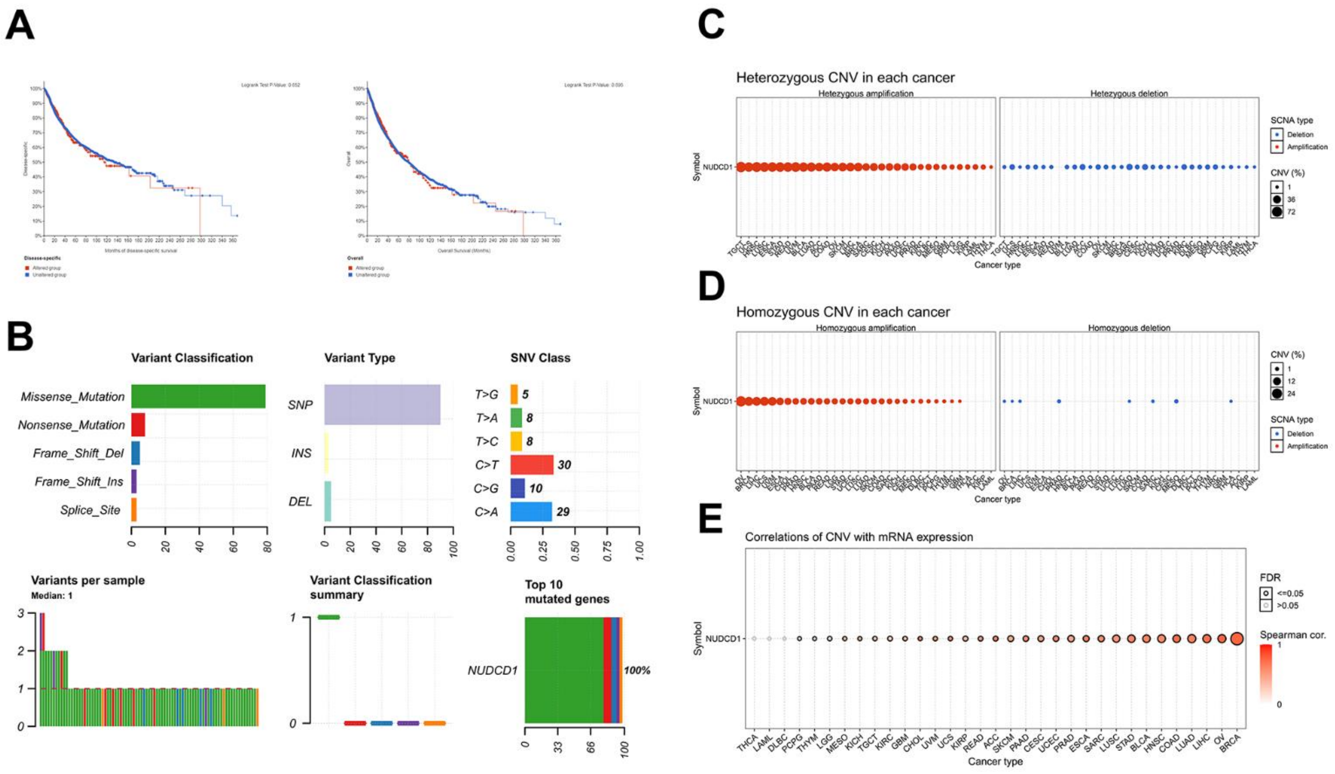

Supplementary Figure 8. SNV and CNV features of NUDCD1 in different tumors. (**A**) Disease-specific survival and overall survival between mutant and wide type of NUDCD1 in human cancers (data from cBioPortal). (**B**) SNV classes of NUDCD1 in each cancer (data from GSCA). The profile of heterozygous CNV (**C**) and homozygous CNV (**D**) of NUDCD1 in each cancer (data from GSCA). (**E**) The correlations between CNV and NUDCD1 mRNA expression in each cancer (data from GSCA).

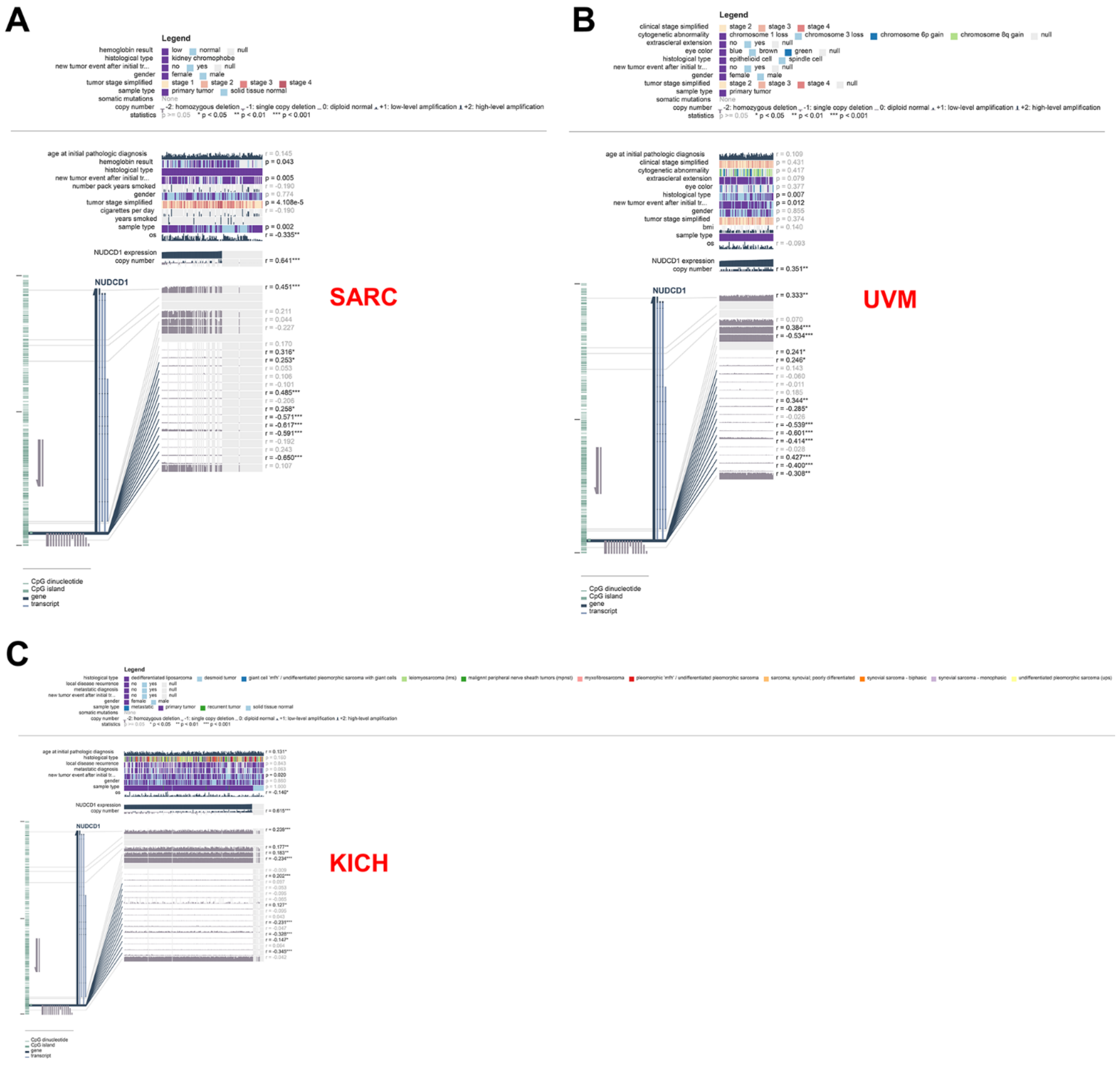

Supplementary Figure 9. The visualization of NUDCD1 expression, methylation and clinical data, as well as the relationships between them in SARC (**A**), UNM (**B**) and KICH (**C**), Data provided by MEXPRESS (https://mexpress.be/).

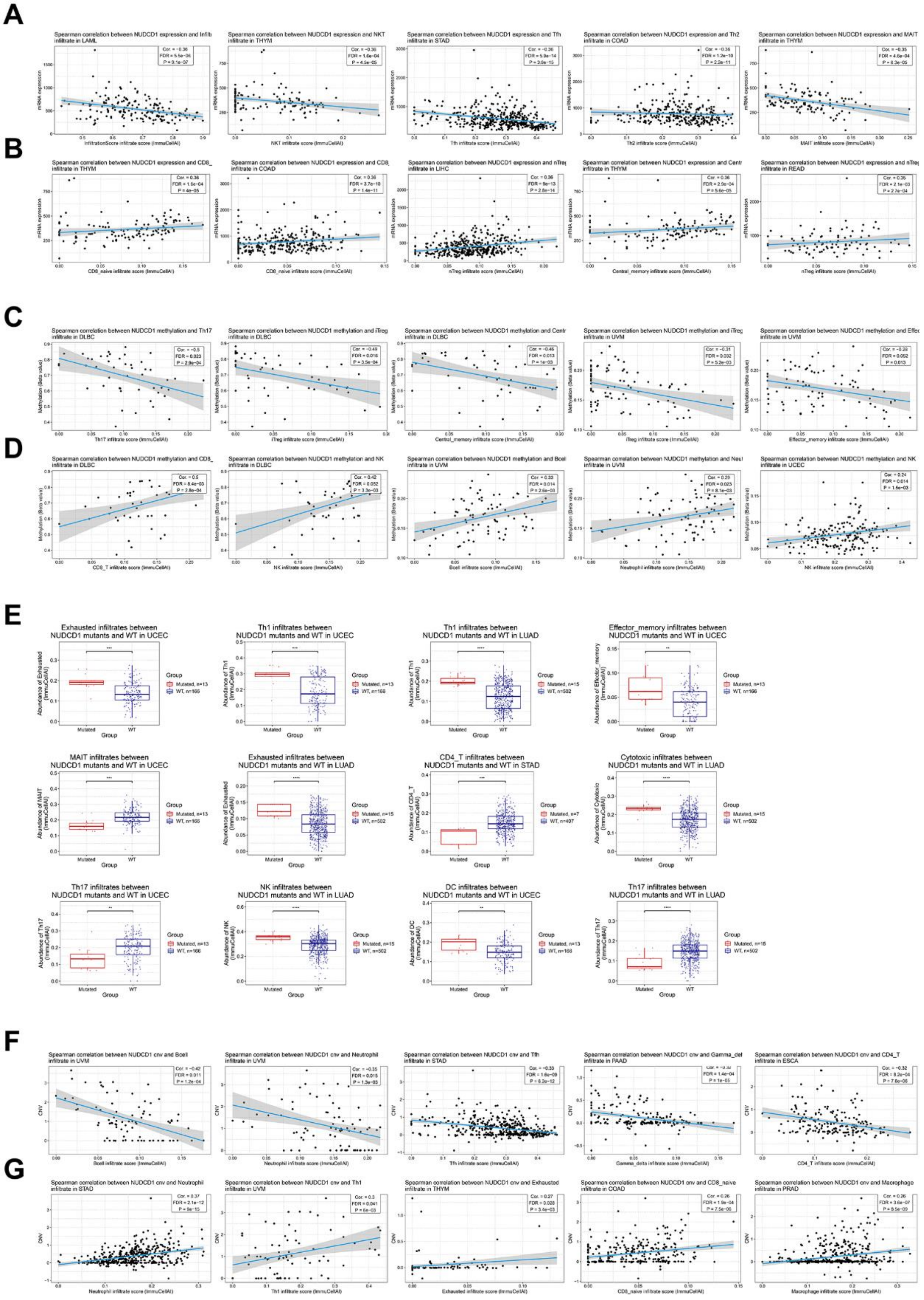

Supplementary Figure 10. The scatterplot data between immune infiltration and NUDCD1 (expression, methylation, SNV, CNV), obtained from GSCA. (**A**) Negative correlation between NUDCD1 expression and immune infiltration in specific cancers (top 5). (**B**) Positive correlation between NUDCD1 expression and immune infiltration in the specific cancers (top 5). (**C**) Negative correlation between NUDCD1 methylation and immune infiltration in specific cancers (top 5). (**D**) Positive correlation between NUDCD1 methylation and immune infiltration in specific cancers (top 5). (**E**) The difference of immune infiltration between mutant and wide type of NUDCD1 in the specific cancers. (**F**) Negative correlation between CNV of NUDCD1 and immune infiltration in specific cancers (top 5). (**G**) Positive correlation between CNV of NUDCD1 and immune infiltration in the specific cancers (top 5).

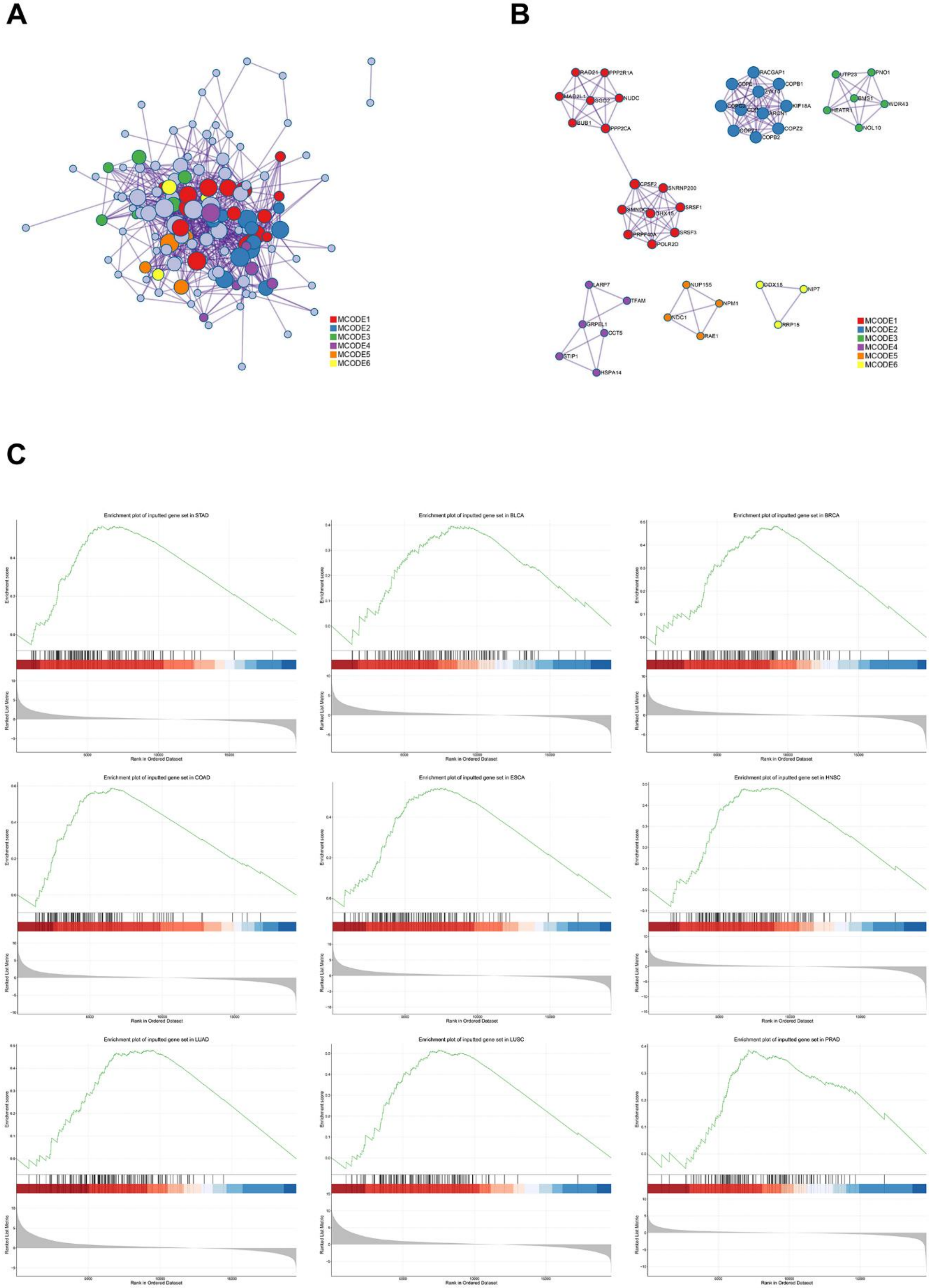

Supplementary Figure 11. Enrichment plot of NUDCD1-related gene set in the detailed cancer types and protein-protein interaction (PPI) analysis for NUDCD1-related partners. (**A**) PPI enrichment analysis of NUDCD1-related gene set. (**B**) Molecular Complex Detection (MCODE) algorithm components identified in the NUDCD1-related gene set. (**C**) Enrichment plot of NUDCD1-related gene set in the detailed cancer types (data from GSCA).

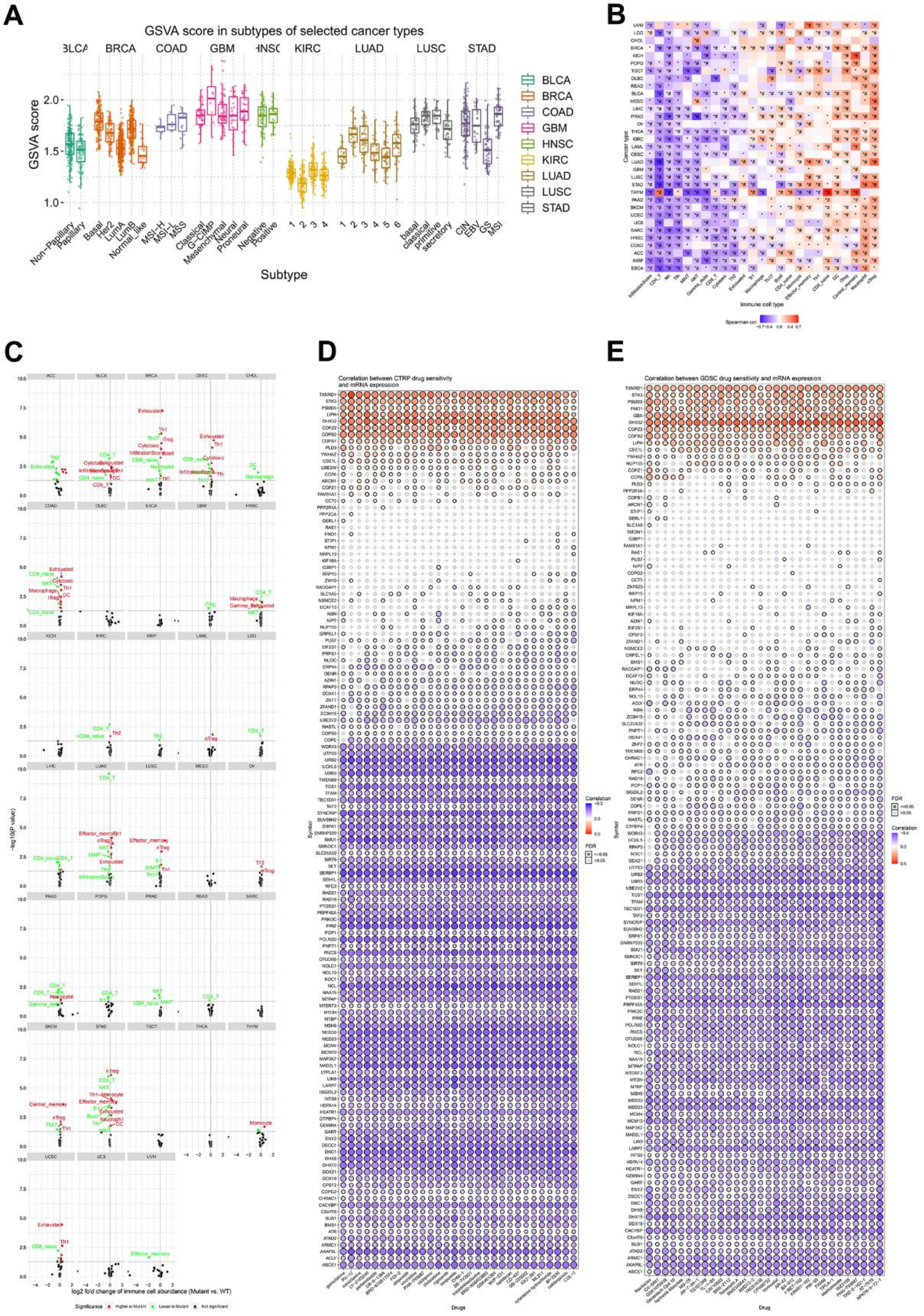

Supplementary Figure 12. GSVA score, immune infiltration, drugs sensitivity analysis for NUDCD1-related genes, obtained from GSCA. (**A**) GSVA score of NUDCD1-related genes in subtypes of detailed cancers. (**B**) Figure summarizes the association between GSVA score of NUDCD1-related genes and immune cell infiltrates in detailed cancers. (**C**) Figure summarizes the difference of immune infiltration between NUDCD1-related genes SNV groups. (**D**) Figure summarizes the correlation between the expression of NUDCD1-related genes and the sensitivity of CTRP drugs (top 30) in pan-cancer. (**E**) Figure summarizes the correlation between the expression of NUDCD1-related genes and the sensitivity of GDSC drugs (top 30) in pan-cancer.
