# Supplementary material for: Identifying the role of NUDCD1 in human tumors from clinical and molecular mechanisms: a study based on comprehensive bioinformatics and experimental validation
Source: Aging (Albany NY). 2023 Jun 19;15(12):5611–49. doi: 10.18632/aging.204813 (PMC10333089; doi:10.18632/aging.204813)
Supplement: Supplementary Tables [file aging-15-204813-s003.pdf]

SUPPLEMENTARY TABLES

Supplementary Table 1. Correlations between NUDCD1 expression and CTRP drug sensitivity.

Symbol	Drug	Cor	Fdr	Entrez
NUDCD1	manumycin A	-0.154035794	0.000043417	84955
NUDCD1	BRD-K41597374	-0.150252194	0.000114327	84955
NUDCD1	indisulam	-0.148117447	0.00015005	84955
NUDCD1	cytarabine hydrochloride	-0.1386582	0.000213329	84955
NUDCD1	AZD7545	-0.14725548	0.000336302	84955
NUDCD1	BRD1812	-0.138545709	0.00042329	84955
NUDCD1	ML311	-0.131533959	0.000456282	84955
NUDCD1	BI-2536	-0.128779639	0.000522686	84955
NUDCD1	clofarabine	-0.128472935	0.000592096	84955
NUDCD1	topotecan	-0.12801365	0.000634888	84955
NUDCD1	erastin	-0.136472614	0.000719367	84955
NUDCD1	methotrexate	-0.135146274	0.000839207	84955
NUDCD1	ruxolitinib	-0.132496393	0.000966762	84955
NUDCD1	pevonedistat	-0.127112242	0.001230478	84955
NUDCD1	COL-3	-0.142978233	0.001570407	84955
NUDCD1	pazopanib	-0.12356731	0.001617376	84955
NUDCD1	linifanib	-0.120727233	0.001728847	84955
NUDCD1	GSK461364	-0.119241143	0.001804143	84955
NUDCD1	3-Cl-AHPC	-0.11847331	0.00206279	84955
NUDCD1	VER-155008	-0.125158113	0.002162812	84955
NUDCD1	phloretin	-0.119533452	0.002164244	84955
NUDCD1	NVP-231	-0.117186071	0.002385234	84955
NUDCD1	CD-437	-0.116076736	0.002499963	84955
NUDCD1	OSI-930	-0.129876311	0.002705571	84955
NUDCD1	teniposide	-0.1589167	0.002920441	84955
NUDCD1	ciclopirox	-0.110125371	0.003434721	84955
NUDCD1	N9-isopropylolomoucine	-0.117950357	0.003437742	84955
NUDCD1	rigosertib	-0.1127841	0.00387617	84955
NUDCD1	sorafenib	-0.118021169	0.004978706	84955
NUDCD1	cerulenin	-0.108437562	0.005098664	84955
NUDCD1	valdecoxib	-0.11089326	0.00549275	84955
NUDCD1	GW-405833	-0.107494574	0.005496153	84955
NUDCD1	KX2-391	-0.106405225	0.005730576	84955
NUDCD1	CIL41	-0.176355564	0.00590742	84955
NUDCD1	brivanib	-0.109167566	0.006470343	84955
NUDCD1	CIL70	-0.151285472	0.006729494	84955
NUDCD1	neopeltolide	-0.231246095	0.007340483	84955
NUDCD1	CAY10618	-0.105032907	0.007469218	84955
NUDCD1	PX-12	-0.101892423	0.00809592	84955
NUDCD1	I-BET151	-0.099606147	0.008437255	84955
NUDCD1	chlorambucil	-0.10298178	0.008672267	84955
NUDCD1	zebularine	-0.103451866	0.008838781	84955
NUDCD1	LY-2183240	-0.098585886	0.009555656	84955
NUDCD1	daporinad	-0.112610134	0.009800851	84955
NUDCD1	SB-225002	-0.09677134	0.010569187	84955
NUDCD1	BRD-K97651142	-0.105110504	0.010642998	84955
NUDCD1	gemcitabine	-0.102685857	0.010696408	84955
NUDCD1	triazolothiadiazine	-0.095545878	0.011237752	84955
NUDCD1	BRD-K35604418	-0.099152675	0.012207327	84955

NUDCD1	AZD7762	-0.096683563	0.012551749	84955
NUDCD1	PAC-1	-0.098339222	0.013960405	84955
NUDCD1	TG-101348	-0.098667763	0.014132771	84955
NUDCD1	nakiterpiosin	-0.09498854	0.014410889	84955
NUDCD1	MST-312	-0.094747204	0.014771387	84955
NUDCD1	obatoclax	-0.094160778	0.015397803	84955
NUDCD1	epigallocatechin-3-monogallate	-0.103578046	0.018456137	84955
NUDCD1	PF-184	-0.092969511	0.01917737	84955
NUDCD1	CIL55A	-0.136645279	0.019798915	84955
NUDCD1	STF-31	-0.094401208	0.019804052	84955
NUDCD1	TW-37	-0.094042971	0.019979531	84955
NUDCD1	decitabine	-0.088670385	0.020212699	84955
NUDCD1	KU-60019	-0.091160951	0.02113365	84955
NUDCD1	elocalcitol	-0.089712075	0.022091147	84955
NUDCD1	fluorouracil	-0.088754489	0.022745177	84955
NUDCD1	BRD-K61166597	-0.091196335	0.023300053	84955
NUDCD1	BRD-K13999467	-0.094691754	0.02497235	84955
NUDCD1	SR-II-138A	-0.084065479	0.025228368	84955
NUDCD1	KHS101	-0.093999949	0.025487741	84955
NUDCD1	BRD-K19103580	-0.103423854	0.025564416	84955
NUDCD1	SMER-3	-0.099086456	0.025728234	84955
NUDCD1	SCH-79797	-0.087643474	0.025955826	84955
NUDCD1	imatinib	-0.092530788	0.027209108	84955
NUDCD1	tigecycline	-0.128664847	0.028414884	84955
NUDCD1	bardoxolone methyl	-0.092212654	0.028647744	84955
NUDCD1	ML239	-0.090653222	0.030536788	84955
NUDCD1	BRD-K66453893	-0.082986933	0.032140481	84955
NUDCD1	MK-1775	-0.084574325	0.033043152	84955
NUDCD1	TPCA-1	-0.084834138	0.033286636	84955
NUDCD1	BRD-K70511574	-0.082754284	0.033467482	84955
NUDCD1	etoposide	-0.082193968	0.033494177	84955
NUDCD1	BRD-K92856060	-0.087614581	0.035031935	84955
NUDCD1	pifithrin-alpha	-0.110748277	0.035788646	84955
NUDCD1	MGCD-265	-0.082172007	0.037392918	84955
NUDCD1	momelotinib	-0.084968769	0.038482427	84955
NUDCD1	BRD-K01737880	-0.352819579	0.03895684	84955
NUDCD1	BRD-K63431240	-0.087170691	0.039797838	84955
NUDCD1	StemRegenin 1	-0.083291153	0.040106979	84955
NUDCD1	trametinib	0.139915727	0.040193597	84955
NUDCD1	parbendazole	-0.077280651	0.042062638	84955
NUDCD1	BRD-K34222889	-0.078640153	0.043376027	84955
NUDCD1	tacrolimus	-0.085286522	0.044801875	84955
NUDCD1	azacitidine	-0.092878126	0.045234613	84955
NUDCD1	LE-135	-0.086219898	0.045711141	84955
NUDCD1	Compound 23 citrate	-0.079492279	0.04645772	84955

Supplementary Table 2. Correlations between NUDCD1 expression and GDSC drug sensitivity.

Symbol	Drug	Cor	Fdr	Entrez
NUDCD1	NPK76-II-72-1	-0.113282875	0.00117523	84955
NUDCD1	AICAR	-0.120947099	0.001557372	84955
NUDCD1	SB590885	0.131335318	0.001813195	84955
NUDCD1	BX-795	-0.121494904	0.002742249	84955
NUDCD1	selumetinib	0.106159698	0.003142449	84955
NUDCD1	Nilotinib	-0.119065023	0.006000703	84955
NUDCD1	PLX4720	0.105259414	0.006023796	84955
NUDCD1	RDEA119	0.097343029	0.006823117	84955
NUDCD1	Camptothecin	-0.105816574	0.008693009	84955
NUDCD1	Cetuximab	0.099980822	0.010501131	84955
NUDCD1	Bleomycin (50 uM)	-0.089152435	0.013449986	84955
NUDCD1	Navitoclax	-0.090778114	0.014729242	84955
NUDCD1	CCT018159	-0.106745799	0.016786475	84955
NUDCD1	MLN4924	-0.119414157	0.017553316	84955
NUDCD1	GW843682X	-0.17258053	0.018349173	84955
NUDCD1	Dabrafenib	0.094317736	0.018969097	84955
NUDCD1	LAQ824	-0.090528237	0.019112171	84955
NUDCD1	ZM-447439	-0.097497964	0.02140719	84955
NUDCD1	TGX221	0.13394576	0.029064306	84955
NUDCD1	SN-38	-0.088083106	0.029673685	84955
NUDCD1	NSC-207895	-0.088373858	0.032206937	84955
NUDCD1	Vorinostat	-0.078086993	0.034760118	84955
NUDCD1	Docetaxel	-0.076695214	0.04189215	84955
NUDCD1	Gefitinib	0.080993211	0.045924902	84955
NUDCD1	TL-1-85	-0.072549249	0.047042309	84955
NUDCD1	YK 4-279	-0.093189683	0.04903313	84955
